# GWAS of Follicular Lymphoma Reveals Allelic Heterogeneity at 6p21.32 and Suggests Shared Genetic Susceptibility with Diffuse Large B-cell Lymphoma

**DOI:** 10.1371/journal.pgen.1001378

**Published:** 2011-04-21

**Authors:** Karin E. Smedby, Jia Nee Foo, Christine F. Skibola, Hatef Darabi, Lucia Conde, Henrik Hjalgrim, Vikrant Kumar, Ellen T. Chang, Nathaniel Rothman, James R. Cerhan, Angela R. Brooks-Wilson, Emil Rehnberg, Ishak D. Irwan, Lars P. Ryder, Peter N. Brown, Paige M. Bracci, Luz Agana, Jacques Riby, Wendy Cozen, Scott Davis, Patricia Hartge, Lindsay M. Morton, Richard K. Severson, Sophia S. Wang, Susan L. Slager, Zachary S. Fredericksen, Anne J. Novak, Neil E. Kay, Thomas M. Habermann, Bruce Armstrong, Anne Kricker, Sam Milliken, Mark P. Purdue, Claire M. Vajdic, Peter Boyle, Qing Lan, Shelia H. Zahm, Yawei Zhang, Tongzhang Zheng, Stephen Leach, John J. Spinelli, Martyn T. Smith, Stephen J. Chanock, Leonid Padyukov, Lars Alfredsson, Lars Klareskog, Bengt Glimelius, Mads Melbye, Edison T. Liu, Hans-Olov Adami, Keith Humphreys, Jianjun Liu

**Affiliations:** 1 Department of Medicine, Clinical Epidemiology Unit, Karolinska Institutet, Stockholm, Sweden; 2 Human Genetics, Genome Institute of Singapore, A*STAR, Singapore, Singapore; 3 Division of Environmental Health Sciences, School of Public Health, University of California Berkeley, Berkeley, California, United States of America; 4 Department of Medical Epidemiology and Biostatistics, Karolinska Institutet, Stockholm, Sweden; 5 Department of Epidemiology Research, Statens Serum Institut, Copenhagen, Denmark; 6 Cancer Prevention Institute of California, Fremont, California, United States of America; 7 Division of Epidemiology, Department of Health Research and Policy, Stanford University School of Medicine, Stanford, California, United States of America; 8 Division of Cancer Epidemiology and Genetics, National Cancer Institute, National Institutes of Health, Bethesda, Maryland, United States of America; 9 College of Medicine, Mayo Clinic, Rochester, Minnesota, United States of America; 10 British Columbia Cancer Research Center, British Columbia Cancer Agency, Vancouver, Canada; 11 Department of Biomedical Physiology and Kinesiology, Simon Fraser University, Burnaby, Canada; 12 Department of Clinical Immunology, University Hospital of Copenhagen, Copenhagen, Denmark; 13 Department of Haematology, Copenhagen University Hospital, Copenhagen, Denmark; 14 Department of Epidemiology and Biostatistics, University of California San Francisco, San Francisco, California, United States of America; 15 Department of Preventive Medicine, Keck School of Medicine, University of Southern California, Los Angeles, California, United States of America; 16 Fred Hutchinson Cancer Research Center, Seattle, Washington, United States of America; 17 University of Washington, Seattle, Washington, United States of America; 18 Department of Family Medicine and Public Health Sciences, Wayne State University, Detroit, Michigan, United States of America; 19 Karmanos Cancer Institute, Detroit, Michigan, United States of America; 20 Division of Etiology, Beckman Research Institute and the City of Hope, Duarte, California, United States of America; 21 Sydney School of Public Health, The University of Sydney, Sydney, Australia; 22 Department of Haematology, St. Vincent's Hospital, Sydney, Australia; 23 University of New South Wales Cancer Research Center, Prince of Wales Clinical School, Sydney, Australia; 24 International Prevention Research Institute, Lyon, France; 25 School of Public Health, Yale University, New Haven, Connecticut, United States of America; 26 Canada's Michael Smith Genome Sciences Centre, British Columbia Cancer Agency, Vancouver, Canada; 27 School of Population and Public Health, University of British Columbia, Vancouver, Canada; 28 Rheumatology Unit, Department of Medicine, Karolinska Institutet and Karolinska University Hospital Solna, Stockholm, Sweden; 29 Institute of Environmental Medicine, Karolinska Institutet, Stockholm, Sweden; 30 Department of Pathology and Oncology, Karolinska Institutet, Stockholm, Sweden; 31 Department of Radiology, Oncology, and Radiation Sciences, Uppsala University, Uppsala, Sweden; 32 Department of Epidemiology, Harvard School of Public Health, Boston, Massachusetts, United States of America; Georgia Institute of Technology, United States of America

## Abstract

Non-Hodgkin lymphoma (NHL) represents a diverse group of hematological malignancies, of which follicular lymphoma (FL) is a prevalent subtype. A previous genome-wide association study has established a marker, rs10484561 in the human leukocyte antigen (HLA) class II region on 6p21.32 associated with increased FL risk. Here, in a three-stage genome-wide association study, starting with a genome-wide scan of 379 FL cases and 791 controls followed by validation in 1,049 cases and 5,790 controls, we identified a second independent FL–associated locus on 6p21.32, rs2647012 (OR_combined_ = 0.64, P_combined_ = 2×10^−21^) located 962 bp away from rs10484561 (r^2^<0.1 in controls). After mutual adjustment, the associations at the two SNPs remained genome-wide significant (rs2647012:OR_adjusted_ = 0.70, P_adjusted_ = 4×10^−12^; rs10484561:OR_adjusted_ = 1.64, P_adjusted_ = 5×10^−15^). Haplotype and coalescence analyses indicated that rs2647012 arose on an evolutionarily distinct haplotype from that of rs10484561 and tags a novel allele with an opposite (protective) effect on FL risk. Moreover, in a follow-up analysis of the top 6 FL–associated SNPs in 4,449 cases of other NHL subtypes, rs10484561 was associated with risk of diffuse large B-cell lymphoma (OR_combined_ = 1.36, P_combined_ = 1.4×10^−7^). Our results reveal the presence of allelic heterogeneity within the HLA class II region influencing FL susceptibility and indicate a possible shared genetic etiology with diffuse large B-cell lymphoma. These findings suggest that the HLA class II region plays a complex yet important role in NHL.

## Author Summary

Earlier studies have established a marker rs10484561, in the HLA class II region on 6p21.32, associated with increased follicular lymphoma (FL) risk. Here, in a three-stage genome-wide association study of 1,428 FL cases and 6,581 controls, we identified a second independent FL–associated marker on 6p21.32, rs2647012, located 962 bp away from rs10484561. The associations at two SNPs remained genome-wide significant after mutual adjustment. Haplotype and coalescence analyses indicated that rs2647012 arose on an evolutionarily distinct lineage from that of rs10484561 and tags a novel allele with an opposite, protective effect on FL risk. Moreover, in an analysis of the top 6 FL–associated SNPs in 4,449 cases of other NHL subtypes, rs10484561 was associated with risk of diffuse large B-cell lymphoma. Our results reveal the presence of allelic heterogeneity at 6p21.32 in FL risk and suggest a shared genetic etiology with the common diffuse large B-cell lymphoma subtype.

## Introduction

**Figure 1:**
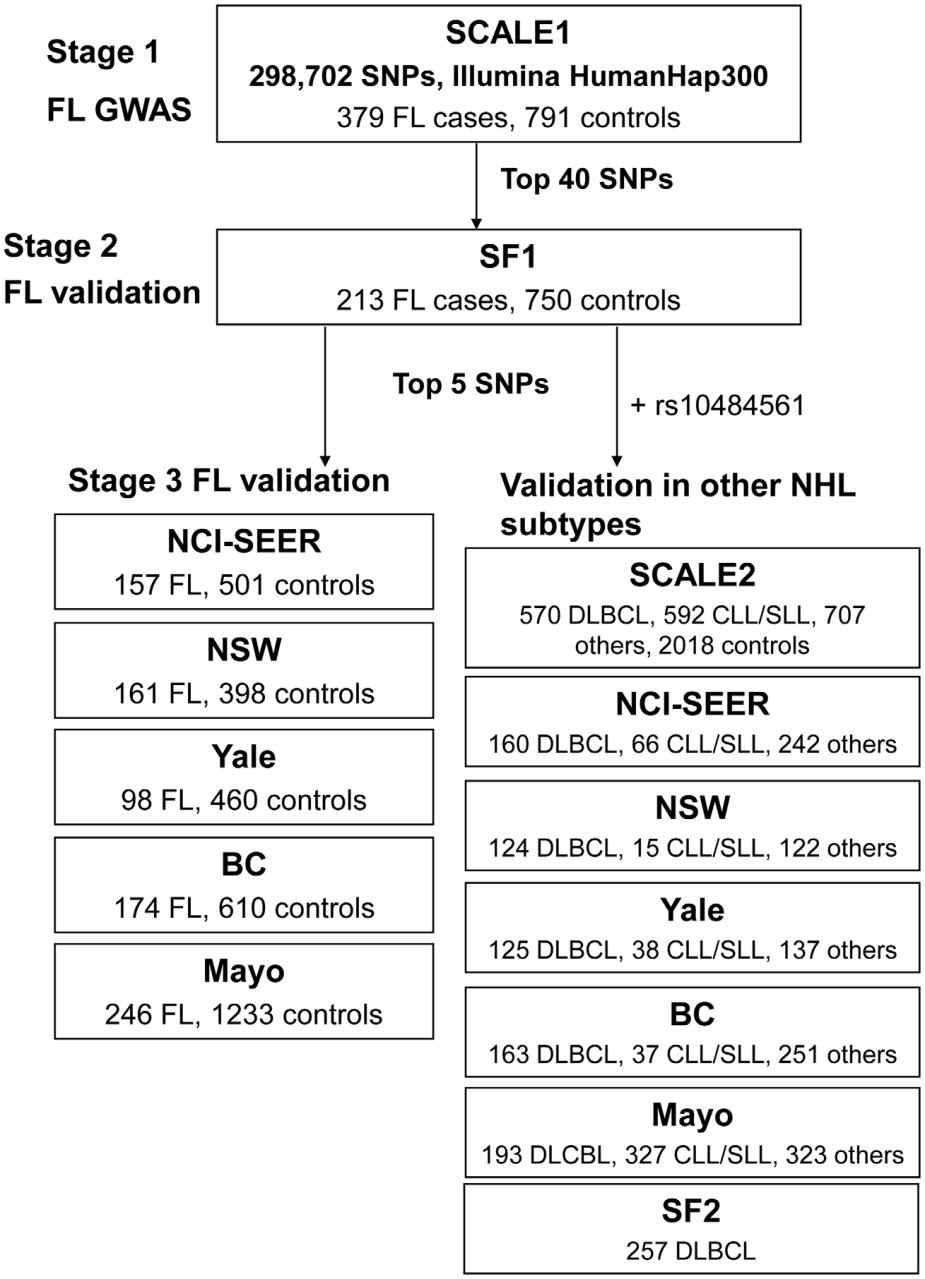
Schematic representation of the three-stage study design. Summary of contributing studies and number of samples per case/control status. Abbreviations: FL: follicular lymphoma, NHL: non-Hodgkin lymphoma, DLBCL: diffuse large B-cell lymphoma, CLL/SLL: chronic lymphocytic leukemia/small lymphocytic lymphoma, SNP: single nucleotide polymorphism, GWAS: genome-wide association study, SCALE: Scandinavian lymphoma etiology, SF: San Francisco, BC: British Columbia, NCI-SEER: National Cancer Institute-Surveillance, Epidemiology and End Results, NSW: New South Wales, Yale: Yale University, Mayo: Mayo Clinic. The complete list of the number of other NHL subtypes in each study is detailed in Table S1.

Non-Hodgkin lymphoma (NHL) represents a diverse group of B- and T-cell malignancies of lymphatic origin. The most common subtypes are of B-cell origin and are further classified on the basis of their resemblance to normal stages of B-cell differentiation [1]. Epidemiological studies indicate that these may have different environmental and genetic risk factors, although some etiological factors may also be shared [2]. Familial studies provide substantial evidence for a genetic influence on susceptibility to the major mature B-cell neoplasms, including diffuse large B-cell lymphoma (DLBCL), follicular lymphoma (FL) and chronic lymphocytic leukemia/small lymphocytic lymphoma (CLL/SLL) [3], [4]. Recent genome-wide association studies (GWAS) of the FL subtype of NHL identified associations with two variants within the human leukocyte antigen (HLA) region, one at 6p21.33 (rs6457327) [5] and the other at 6p21.32 (rs10484561) [6]. Additional true associations, particularly in the HLA region, may have been missed because a limited number of samples were used in the initial genome-wide screens, and the selection of a few top single nucleotide polymorphisms (SNPs) for validation is further subject to chance. In this study, we conducted a larger independent genome-wide scan of FL using 379 cases and 791 controls from the Scandinavian Lymphoma Etiology (SCALE) study of Sweden and Denmark, which was used in the validation of the previous GWAS [6]. This scan was followed by two stages of validation in European-ancestry cases of FL and other common B-cell NHL subtypes and controls from the US, Canada and Australia (Table 1, Table S1, Table S2, Figure 1).

**Table 1:** Summary of contributing studies, genotyping methods, and number of samples per case/control status.

Stage	Study	Population	Genotyping method	Controls	FL	Other NHL subtypes*
1	SCALE1	Denmark/Sweden	Illumina 317k	791	379	-
2	SF1	San Francisco Bay Area, USA	Illumina HumanCNV370-Duo Beadchip	750	213	-
3/subtype validation	NCI-SEER	USA	Taqman	501	157	468
	NSW	New South Wales, Australia	Taqman	398	161	261
	Yale	USA	Taqman	460	98	300
	BC	British Columbia, Canada	Taqman	610	174	451
	Mayo	Minnesota, Iowa, Wisconsin, USA	OPA	1233	246	843
	SCALE2	Denmark/Sweden	Sequenom/Taqman	2018	-	1869
	SF2	San Francisco Bay Area, USA	Illumina HumanCNV370-Duo Beadchip	-	-	257
**Total**				**6761**	**1428**	**4449**

Abbreviations: SCALE: Scandinavian lymphoma etiology, SF: San Francisco, NCI-SEER: National Cancer Institute- Surveillance, Epidemiology and End Results, NSW: New South Wales, Yale: Yale University, BC: British Columbia, Mayo: Mayo Clinic, OPA: oligonucleotuide pool assays (Illumina GoldenGate).

*The complete list of the number of other NHL subtypes in each study is detailed in Table S1.

## Results

**Figure 2:**
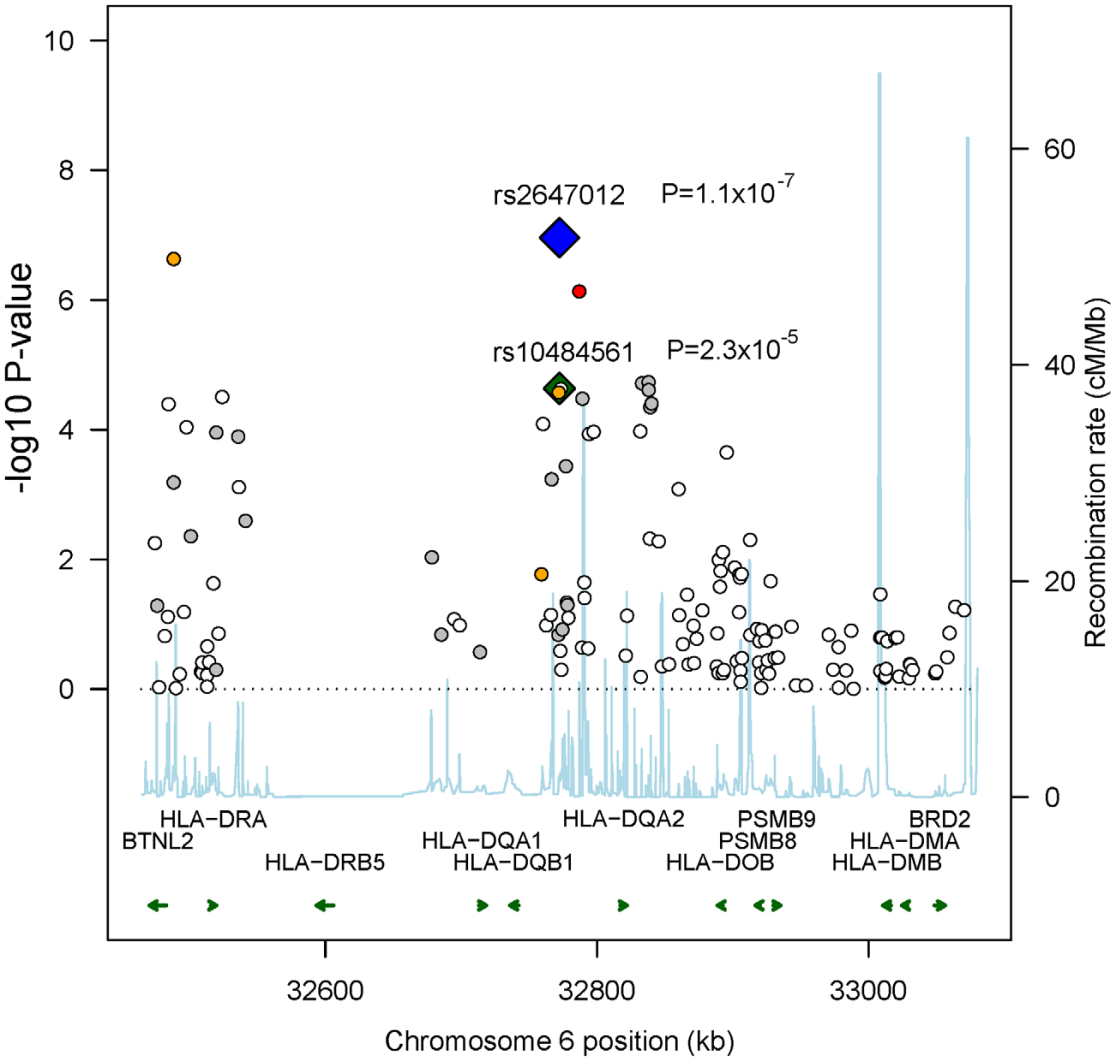
Recombination plot showing associations in 6p21.32 in Stage 1. Plot showing the pattern of associations in Stage 1, the recombination rate (build 36, HapMap CEU) and genes located in the region. The two SNPs showing independent association and their respective P-values are labeled (blue: rs2647012, green: rs10484561); other SNPs are color-coded according to their LD with rs2647012 (red r^2^>0.8, orange 0.5–0.8, grey 0.2–0.5, white <0.2).

In total, 298,168 SNPs were analyzed in Stage 1 (λ = 1.028; λ_1000_ = 1.055 [7]), in which we observed suggestive associations (adjusted trend P-value<10^−5^) at 4q32.3, 6p21.32 and 10q25.3 (Table S3) with the strongest at rs2647012 (odds ratio (OR) = 0.58, P_PCAadjusted_ = 1.59x10^−7^) within the HLA class II region on 6p21.32. Sixteen SNPs in close proximity to the *HLA-DQ* genes showed association with adjusted P-values<10^−4^, including the previously reported rs10484561 (Figure 2, Table S4) [6]. The previously reported HLA class I associated SNP rs6457327 [5] was modestly associated with FL risk (OR = 0.82, P = 0.03) in Stage 1, and was not in linkage disequilibrium (LD; r^2^ = 0) with any of the top 100 SNPs.

In Stage 2, we carried out an *in silico* validation of the top 40 SNPs from Stage 1 (Table S5) in 213 FL cases and 750 controls from the San Francisco Bay Area, USA (Table 1), the study that reported an association at 6p21.32 [6]. Among 38 out of 40 SNPs, seven showed association (P<0.05) in Stage 2 (Table S5), six of which were located within the 6p21.32 region. We tested the independence of multiple association signals in 6p21.32 using a stepwise logistic regression analysis (entering SNPs based on a criterion of likelihood ratio test p-value<0.05) and found that with rs2647012 (the top SNP within the region) forced in the model, only the addition of rs10484561 contributed significantly to the association with increased risk of FL. The OR for this SNP, adjusted for rs2647012, was 1.43, P = 0.006 (Table S6).

**Figure 3:**
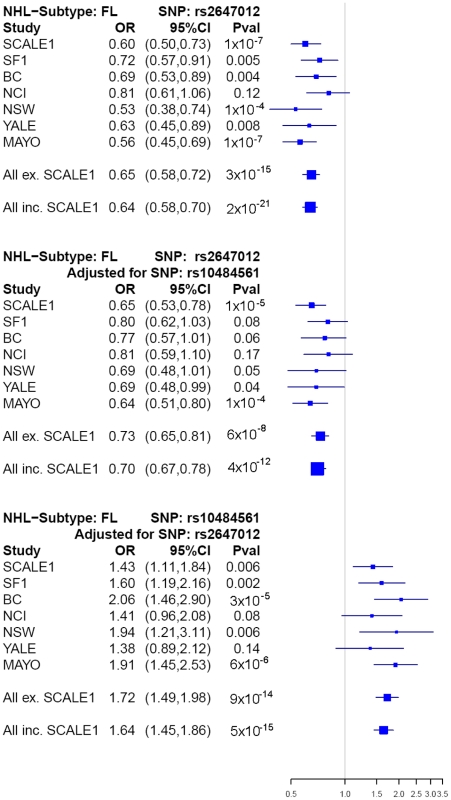
Forest plots of main associations with risk of FL. Forest plots showing the associations in each study (ORs and P-values) at rs2647012 before adjustment (P_heterogeneity_ = 0.32), and at rs2647012 (P_heterogeneity_ = 0.67) and rs10484561 (P_heterogeneity_ = 0.54) after mutual adjustment. Squares indicate the odds ratios, with the size proportional to the weight of the study in the meta-analysis. Abbreviations: CI: confidence interval, SCALE: Scandinavian lymphoma etiology, SF: San Francisco, BC: British Columbia, NCI: National Cancer Institute-Surveillance, Epidemiology and End Results, NSW: New South Wales, YALE: Yale University, MAYO: Mayo Clinic.

After excluding previously identified and non-independent association signals, we selected rs2647012, and an additional four top SNPs to be taken forward to a third stage (Table S7, S8), wherein these were genotyped in 836 FL cases and 3202 controls from the Mayo Clinic (US) [8], National Cancer Institute-Surveillance, Epidemiology and End Results (NCI-SEER, US) [9], Yale University (US) [10], New South Wales (NSW, Australia) [11] and British Columbia (BC, Canada) [12] studies. The association of rs2647012 with FL was validated, showing consistent associations with similar ORs (no heterogeneity, P = 0.32) across all independent studies and reaching genome-wide significance in both the combined analysis of the validation samples (P = 3×10^−15^) and the combined analysis of all three stages (1428 FL cases, 4743 controls; OR = 0.64, P = 2×10^−21^) (Table.2, Figure 3). After adjustment for rs10484561, the association at rs2647012 remained genome-wide significant with minimal change in magnitude (OR_adjusted_ = 0.70, P_adjusted_ = 4×10^−12^). The LD between the two SNPs is low (r^2^<0.1 in the SCALE controls and HapMap CEU [Utah residents with northern and western European ancestry] samples release27). Taken together, our results suggest that the association at rs2647012 is independent from rs10484561, and tags a different disease-predisposing variant. We also found suggestive evidence for an association at rs6536942 on 4q32.3 (OR = 1.36, P = 2×10^−5^) (Table 2, Figure S1A).

**Table 2:** Summary of main findings in genome-wide association study (GWAS) and validation stages in risk* of follicular lymphoma (FL), diffuse large B-cell lymphoma (DLBCL), or marginal zone lymphoma (MZL), per study and combined.

	FL				DLBCL	MZL
	rs2647012	rs2647012adj for rs10484561**	rs10484561adj for rs2647012**	rs6536942	rs10484561	rs2647012
	chr6:32772436 *HLA-DQB1*			chr4:167205644 *TLL1*	chr6:32773398 *HLA-DQB1*	chr6:32772436 *HLA-DQB1*
**Stage 1**						
SCALE1	1×10^−7^	1×10^−5^	0.006	1×10^−6^	-	-
	0.60 (0.50–0.73)	0.65 (0.53–0.79)	1.43 (1.11–1.84)	1.86 (1.45–2.34)		
**Stage 2**						
SF	0.005	0.08	0.002	-	0.04	-
	0.72 (0.57–0.91)	0.80 (0.62–1.03)	1.60 (1.19–2.16)		1.36 (1.05–1.80)	
**Stage 3**						
BC	0.004	0.06	3×10^−5^	0.72	0.001	0.01
	0.69 (0.53–0.89)	0.77 (0.58–1.01)	2.06 (1.46–2.90)	1.08 (0.72–1.61)	1.77 (1.26–2.48)	1.56 (1.10–2.22)
NCI-SEER	0.12	0.17	0.08	0.13	0.15	0.06
	0.81 (0.61–1.06)	0.81 (0.59–1.10)	1.41 (0.96–2.08)	1.34 (0.92–1.95)	1.32 (0.90–1.94)	1.51 (0.99–2.31)
NSW	1×10^−4^	0.05	0.006	0.42	0.01	0.45
	0.53 (0.38–0.73)	0.69 (0.48–1.01)	1.94 (1.21–3.11)	1.21 (0.76–1.91)	1.74 (1.12–2.72)	1.23 (0.72–2.08)
Yale	0.008	0.04	0.14	0.74	0.40	0.73
	0.63 (0.45–0.89)	0.69 (0.48–0.99)	1.38 (0.90–2.12)	1.08 (0.67–1.74)	1.18 (0.79–1.77)	1.10 (0.63–1.92)
Mayo	1×10^−7^	1×10^−4^	0.28	0.28	0.01	0.38
	0.56 (0.45–0.69)	0.64 (0.51–0.80)	1.17 (1.45–2.53)	1.17 (0.88–1.56)	1.52 (1.11–2.08)	1.18 (0.82–1.69)
SCALE2	-	-	-	-	0.08	0.10
					1.19 (0.98–1.44)	1.29 (0.95–1.74)
Joint	3×10^−15^	6×10^−8^	9×10^−14^	0.06	-	-
	0.65 (0.58–0.72)	0.73 (0.65–0.82)	1.72 (1.49–1.98)	1.18 (1.00–1.40)		
**All** (Stages 1,2,3)	2×10^−21^	4×10^−12^	5×10^−15^	2×10^−5^	1×10^−7^	6×10^−4^
	0.64 (0.58–0.70)	0.70 (0.67–0.78)	1.64 (1.45–1.86)	1.36 (1.18–1.56)	1.36 (1.21–1.52)	1.32 (1.13–1.55)
P_heterogeneity_ †	0.32	0.67	0.54	0.09	0.28	0.83

*The trend test P-value for each study is shown with the corresponding odds ratios (OR) and confidence interval (CI) below.

**Additional adjustment for rs6457327, rs4947332, rs1794265, rs1800629, rs2517448 did not confer other than marginal changes.

†Test of heterogeneity carried out assuming heterogeneity of effect of the “other” SNP.

SCALE: Scandinavian Lymphoma Etiology; SF: San Francisco; BC: British Columbia; NCI-SEER: National Cancer Institute-Surveillance, Epidemiology and End Results; NSW: New South Wales.

To fine-map the association signals in the HLA class II region, we imputed 10,639 SNPs within 600 kb surrounding the top SNP rs2647012 using data from the 1000 Genomes (1000G, 60 CEU subjects, August 2009) and HapMap projects (HapMapII release 22, CEU) in Stage 1. Among the imputed SNPs, 258 SNPs located in a strong LD block of 236 kb (r^2^>0.8) showed stronger evidence of association than all the genotyped SNPs within the region (Figure S2). Since a moderate discordance of reference genotypes was observed between 1000 G and HapMapII, we analyzed only SNPs showing a concordance of >95% in the two datasets and identified the strongest association at rs9378212 (OR = 1.66, P = 3.21×10^−8^), located 219 kb upstream of rs2647012 (r^2^ = 0.56 in controls). We subsequently confirmed the imputed genotypes by Taqman genotyping in 345 of the FL case subjects used in Stage 1 and found a 99.4% concordance with the imputed genotypes, demonstrating high confidence in the results of the imputation.

**Figure 4:**
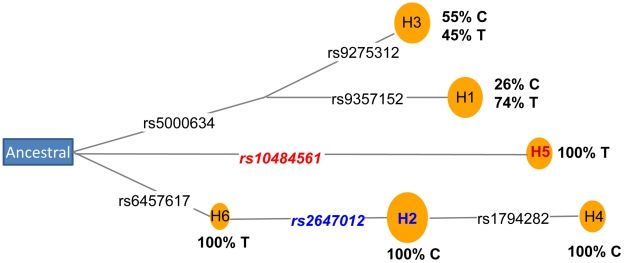
Coalescence analysis of rs2647012 and rs10484561. Median-joining network [14] of haplotypes constructed using seven SNPs (Table S9). Circles represent haplotypes with area proportional to their frequency. SNPs are shown on the links (black lines). SNPs and haplotypes associated with increased or decreased FL risk are labeled in red or blue, respectively. The percentage of alleles of the imputed SNP rs9378212 (C/T) phased on each haplotype are shown in bold.

Next, we performed a haplotype analysis using rs2647012, rs10484561 and an additional 12 adjacent genotyped SNPs located within a block of minimal recombination. Out of the eight haplotypes identified, three were neutral (OR = 0.9–1.1), three increased risk (ORs>1.2; strongest risk haplotype tagged by rs10484561) and two were protective (OR≤0.8; both tagged by rs2647012) (Table S9), suggesting the presence of at least two susceptibility alleles within the region. Coalescence analysis of the eight haplotypes indicated that rs2647012 and rs10484561 arose on two distal branches of the ancestral recombination graph [13] (Figure S3), which was also supported by the analysis of median-joining network [14] using seven SNPs without any recombination (Figure 4). Further haplotype analysis of the seven genotyped SNPs (Table S9) and the imputed SNP rs9378212 indicated that the two alleles of rs9378212 tag the two different evolutionary lineages (Figure 4), each harboring either rs2647012 or rs10484561. Thus, the associations at the two SNPs are likely due to two distinct susceptibility variants, instead of a single risk allele, that arose independently on different haplotype backgrounds.

The FL-associated SNP, rs10484561, was previously found to tag the extended haplotype HLA-DQA1*0101-HLA-DQB1*0501-HLA-DRB1*0101 [6]. Here, to test whether any HLA class II alleles may also be responsible for the observed association at rs2647012, we imputed known HLA tag SNPs [15], [16] using data from the 1000G and HapMapII European datasets. We confirmed the association of the HLA-DRB1*0101-HLA-DQA1*0101-HLA-DQB1*0501 extended haplotype, tagged by rs10484561. The association at rs2647012 remained significant after adjustment for these three HLA alleles (OR = 0.64, P = 8.11×10^−6^), suggesting that these are not driving the association at rs2647012. Furthermore, rs2647012 was not in strong LD (r^2^<0.8 in HapMap CEU or SCALE controls) with any other known HLA tags [15], including those tagging FL-associated alleles previously reported [17], [18] (r^2^<0.39 with the six HLA-DRB1*13 tag SNPs [rs2395173, rs2157051, rs4434496, rs6901541, rs424232, rs2050191] [17] and r^2^<0.25 with the three HLA-B*0801 and HLA-DRB*0301 tag SNPs [rs6457374, rs2844535, rs2040410] [15]). Of the other 17 HLA class II alleles (∼39% of all the class II alleles) that could be imputed, none showed significant association or were found to be responsible for the association at rs2647012 (Table S10). Detailed HLA allelotyping on large numbers of cases and controls is needed to determine if particular HLA class II alleles are responsible for the observed association at rs2647012.

To assess whether the FL-associated SNPs may be involved in the development of other NHL subtypes, we genotyped the five SNPs selected for Stage 3 together with rs10484561 in a total of 1592 DLBCL, 1075 CLL/SLL, 336 marginal zone lymphoma (MZL), 262 mantle cell lymphoma, 306 T-cell lymphoma and 878 rare or unspecified NHL cases and 5220 controls from the SCALE2, SF2, BC, Mayo, NCI-SEER, Yale and NSW studies (Table 1, Table S1, Figure 1). Among these SNPs, rs10484561 showed evidence of association with DLBCL (OR = 1.36, P = 1.41×10^−7^) (Figure S1B) and all NHL (OR = 1.23, P = 6.81×10^−7^). ORs were consistent across the seven studies. There was also a suggestive association for rs2647012 with MZL (OR = 1.32, P = 6.34×10^−4^) (Table.3), consistent across six studies.

Finally, we investigated the possibility of additional susceptibility loci for FL outside of the HLA region by performing a joint analysis of the top 41 to 1000 variants of our scan and the previously published GWAS of follicular lymphoma [6]. From this combined analysis, we did not find any additional markers with a strong association (P<10^−6^) with FL that were not in LD with our top 5 markers taken forward to stage 3 (data not shown).

**Table 3:** Meta-analysis of associations between rs1048456, the top 5 markers, and non-Hodgkin lymphoma (NHL) subtypes (including follicular lymphoma [FL], diffuse large B-cell lymphoma [DLBCL], chronic lymphocytic leukemia/small lymphocytic lymphoma [CLL/SLL], and others) and overall (All NHL).

	FL*	DLBCL	CLL/SLL	Marginal zone	Mantle cell	T-cell	All NHL*
Marker	*P*-value	*P*-value	*P*-value	*P*-value	*P*-value	*P*-value	*P*-value
	OR (95% CI)	OR (95% CI)	OR (95% CI)	OR (95% CI)	OR (95% CI)	OR (95% CI)	OR (95% CI)
**rs10484561**	1.06×10^−21^	1.41×10^−7^	0.05	0.31	0.54	0.22	6.81×10^−7^
chr6:32773398 *HLA-DQB1*	1.94 (1.70–2.21)	1.36 (1.21–1.52)	0.85 (0.73–1.00)	0.87 (0.66–1.14)	0.91 (0.68–1.22)	0.84 (0.64–1.12)	1.23 (1.13–1.34)
**rs2647012**	6.53×10^−16^	0.36	0.12	6.34×10^−4^	0.15	0.04	0.02
chr6:32772436 *HLA-DQB1*	0.65 (0.58–0.72)	0.96 (0.88–1.05)	0.92 (0.84–1.02)	1.32 (1.13–1.55)	1.14 (0.95–1.36)	1.20 (1.01–1.41)	0.94 (0.89–0.99)
**rs6536942**	0.06	0.22	0.53	0.28	0.51	0.56	0.07
chr4:167205644 *TLL1*	1.18 (1.00–1.40)	1.09 (0.95–1.24)	1.05 (0.90–1.22)	1.14 (0.90–1.46)	0.91 (0.68–1.22)	1.08 (0.84–1.40)	1.08 (0.99–1.18)
**rs9277554**	0.08	0.42	0.20	0.99	0.08	0.18	0.93
chr6:33163516 *HLA-DPB1*	0.91 (0.81–1.01)	0.96 (0.87–1.06)	1.07 (0.96–1.19)	1.00 (0.84–1.19)	1.19 (0.98–1.44)	1.13 (0.94–1.36)	1.00 (0.94–1.06)
**rs441890**	0.91	0.14	0.74	0.47	0.63	0.95	0.37
chr8:71727221 *LACTB2*	1.01 (0.90–1.13)	1.07 (0.98–1.17)	1.02 (0.92–1.12)	1.06 (0.90–1.25)	0.96 (0.80–1.15)	0.99 (0.84–1.18)	1.03 (0.97–1.09)
**rs716183**	0.84	0.78	0.35	0.59	0.94	0.80	0.49
chr10:118894485*VAX1*	0.99 (0.88–1.10)	0.99 (0.90–1.08)	0.95 (0.87–1.05)	1.05 (0.89–1.23)	1.01 (0.84–1.20)	0.98 (0.83–1.16)	0.98 (0.93–1.04)

*Excluding SCALE1 for FL.

OR: odds ratio, CI: confidence interval.

## Discussion

Through the identification of a second variant, rs2647012, that is independent of the previously identified risk variant rs10484561 [6] within the 6p21.32 region, our findings substantiate a major link between HLA class II loci and genetic susceptibility to FL. In addition, our study revealed evidence that rs10484561 is associated with DLBCL risk suggesting some shared biological mechanisms of susceptibility between these two common NHL subtypes. The association of rs2647012 with FL risk was not detected in earlier GWAS studies [5], [6], and that of rs10484561 with DLBCL risk previously reported was only marginal [6], perhaps because of the smaller sample sizes in Stage 1. The number of FL cases scanned in this study was almost double compared to the previous individual GWAS [6].

HLA class II molecules are expressed in antigen presenting cells such as B-lymphocytes, and act to present exogenous antigens to CD4+ helper T-cells. Efficiency of antigen presentation may influence lymphomagenesis through effects on anti-tumor immunity or on immune response to infections that are directly or indirectly oncogenic (e.g., through viral genome insertion or nonspecific chronic antigenic stimulation) [19]. Allelic variants in coding regions may affect the structure of the peptide binding groove of the class II molecules, leading to differences in the efficiency of oncogenic peptide binding or T-cell recognition. Coding sequence variation in the molecules encoded by the extended HLA-DRB1*0101-HLA-DQA1*0101-HLA-DQB1*0501 haplotype may be responsible for the association at rs10484561 [6].

Alternatively, variants in the regulatory sequences may influence the expression level of the HLA molecules and consequently the efficiency of antigen presentation. We note that rs2647012 is strongly associated with the average expression levels of *HLA-DRB4* (β = 0.78, P = 3.4×10^-22^) and *HLA-DQA1* (β = -0.58, P = 5.1×10^−13^) probes in Epstein-Barr virus-transfected lymphoblastoid cell lines (mRNA by SNP browser) [20], and rs10484561 is also associated with the expression levels of *HLA-DQA1* probes (β = -0.884, P = 1.6×10^−10^). We speculate that this may be an alternative mechanism underlying the observed associations, especially at rs2647012.

Interestingly, SNPs within the same LD block harboring rs2647012 (r^2^>0.7 in HapMap CEU) have previously been associated with rheumatoid arthritis with the same direction of effect [21]. Since autoimmune disorders such as rheumatoid arthritis and Sjögren syndrome are associated with increased risk of NHL, in particular with DLBCL but also with FL [22], our finding may suggest a molecular link between these diseases, although their associations within this region of high LD could also be due to different causal variants.

Previously, large-scale candidate gene studies have pointed to susceptibility loci in the HLA class III region mainly between the *TNF* variant *–*308G->A (rs1800629) and risk of DLBCL [23], [24]. We provide novel evidence of association of DLBCL with an independent HLA marker in the class II region (rs10484561; r^2^ = 0), 1.1Mb away from rs1800629, strongly suggesting that alleles in the HLA class II region may play an important role in the pathogenesis of this subtype as well. The weaker association of rs10484561 with DLBCL (OR 1.36) than with FL (OR 1.95) [6] could imply that the DLBCL-association is confined to a subset of DLBCL tumors with specific morphological or molecular features more closely related to FL, such as the germinal center-like B-cell phenotype [25]. However, the observed effects could also be due to modification of other concurrent DLBCL-specific susceptibility variants, or rs10484561 could tag a more strongly associated marker in this region of high LD.

Moreover, we found suggestive evidence of association at rs6536942 on 4q32.3, located within an intron of the tolloid-like 1 (*TLL1*) gene, with FL risk. However, larger studies are needed to validate this finding. Although the strongest associations so far have been observed in the HLA region, and extended pooling of available scan data failed to identify additional loci outside of HLA, we expect that future larger meta-GWAS efforts will more robustly identify additional loci in other regions.

In conclusion, our results strongly suggest that future genetic and functional work focused on the HLA class II region will provide important insight into the disease pathology of FL, DLBCL and other subtypes of NHL. In addition, further studies of this region and potential interaction with environmental factors in NHL risk, and of NHL prognosis are warranted.

## Methods

### Ethics statement

The studies described in this manuscript have been approved by the ethics committee of the respective institutions: Karolinska Institutet (Sweden), Scientific Ethics Committee system (Denmark), University of California, Berkeley (US), National Cancer Institute, National Institutes of Health (US), Mayo Clinic (US), University of British Columbia (Canada), Yale University (US), University of Sydney (Australia).

### Study subjects

The SCALE study is a population-based study of the etiology of NHL carried out in all of Denmark and Sweden during 1999 to 2002 [26]. NHL subtype diagnoses were reviewed and reclassified according to the World Health Organization (WHO) classification [1] as previously described [26]. For this GWAS (SCALE1) we used DNA from 400 cases with follicular lymphoma (FL; 150 from Denmark and 250 from Sweden) and from 150 Danish controls, individually matched to the Danish FL cases by sex and age at study inclusion. We also used material collected from 673 control subjects in a separate Swedish population-based case-control study of rheumatoid arthritis (the Eira study) [21], [27]. The latter was conducted during 1996 to 2005 among residents 18 to 70 years of age in the southern and central parts of Sweden (including 90% of Swedish residents). Hence, the population controls recruited in this study were considered to represent the same study population as the Swedish component of the SCALE study with regard to genetic variation. Genotyping completion rates were similar between cases and controls; out of 400 cases and 823 controls genotyped, 379 cases (95%) and 791 controls (96%) were included in the final analysis. Study subjects used in Stages 2, 3 and validation in other NHL subtypes (Table 1, Table S1, S2) have been previously described [6], [8]–[12], and details are available as supporting text (Text S1). For the SCALE2 NHL subtype validation study, we used the rest of the lymphoma cases with blood samples originally recruited in SCALE (n = 1869), Danish control subjects not included in the GWAS (n = 556), a second set of control subjects from the Eira study (n = 742) and a third group of controls recruited in a national population-based case-control study of breast cancer, the Cancer and Hormones Replacement in Sweden (CAHRES) study [28] (n = 720). The control subjects from this study were randomly selected from the Swedish general population to match the expected age distribution of the participating breast cancer cases (50 to 74 years).

### Genotyping

Stage I genotyping of 317,503 single nucleotide polymorphisms (SNPs) was done on the HumanHap300 (version 1.0) array. Validation genotyping was done using Sequenom iPlex; SNPs in the human leukocyte antigen (HLA) region that failed primer design for Sequenom assays were genotyped using Taqman (Applied Biosystems).

### Genome-wide association study

The scan included 317,503 SNPs from the HumanHap300 (version 1.0) array. The datasets were filtered on the basis of SNP genotyping call rates (≥>95% completeness), sample completion rate (≥90%), minor allele frequency (MAF; all subjects as well as cases and controls separately ≥0.03) and non-deviation from Hardy-Weinberg equilibrium (HWE; p<10^−6^). We also excluded SNPs with cluster plot problems, and those on the X and Y chromosomes. Study subjects with gender discrepancies and/or labelling errors were removed. We also removed individual samples with evidence of cryptic family relationships (identified using the–genome command in PLINK). To detect outliers in terms of population stratification, we performed principal component (PC) analysis using the EIGENSTRAT software (Figure S4). A subset of linkage disequilibrium (LD) thinned SNPs was selected such that all pair-wise associations had r^2^<0.2, and long-range regions of high LD, reported to potentially confound genome scans, were removed [29]. Twenty-five samples were removed as population outliers on the basis of their values on the first three PCs. To adjust for possible stratification in our association analyses we adjusted the regression analyses using the first three PCs; the number of PCs used for adjustment was determined by plotting the eigenvalues and locating the position of the “elbow” on the scree plot (Figure S5). Wald tests, treating minor allele counts as continuous covariates were used to test for association. The genomic inflation factor (λ) was calculated to be 1.0283 after adjusting for the first three PCs, suggesting the presence of minimal stratification. Quantile-quantile plots for the associations before and after adjustment are shown in Figure S6. Finally, we assessed associations of age and sex with main genotypes among the control subjects to address the possibility of confounding by these factors (Table S11). As there was no evidence of associations of age or sex with genotypes among the controls, we did not adjust for them in the final main effects analyses of genotypes.

### Validation and meta-analysis

In Stage 2, similar quality control measures were applied as in Stage 1, including genotyping call rate ≥95%, sample completion rate ≥90%, and MAF ≥0.05. We tested each validation study for association using trend tests. For meta-analyses across studies and NHL subtypes, we used the Cochran-Mantel-Haenszel method to calculate the combined odds ratio and P-value, and χ^2^ tests for heterogeneity. Multivariate logistic regression was used to test for independence of SNP effects. For validation among other NHL subtypes, the control subjects were the same as those in Stages 2 and 3 for validation in FL for all studies except SCALE2. Only European-ancestry subjects were included, and the possibility of population stratification affecting the results has been thoroughly explored and found to be low in earlier investigations in the same populations [6], [8].

### Imputation

We used IMPUTEv1 for the imputation of SNPs from the 1000 Genomes pilot1 CEU data (August 2009 release); and the HapMap Phase II release 22 CEU data. We set a strict threshold for imputation, using only SNPs with confidence scores of ≥0.9, call rates ≥90%, non-deviation from Hardy-Weinberg equilibrium P >0.001 and MAF >0.01. The imputation was done on the Stage 1 samples separately for each of the two reference datasets and SNPs showing a discordance of >5% between the genotypes imputed with the two datasets were excluded from further analysis. The data were then merged using HapMap II as the master dataset to which additional imputed SNPs from the 1000 Genomes dataset were added. HLA alleles were imputed by identifying tag SNPs [15] from the genotyped and imputed SNP dataset. We used PLINK for haplotype imputation with the tag SNPs and downstream association analyses. Only haplotypes with call rates >90%, MAF>1% and probability thresholds >0.8 were analyzed.

### Haplotype and coalescence analyses

For coalescence analysis all 12 SNPs (genotyped in this study and within a region of ∼177 Kb) adjacent to the two SNPs associated with the FL risk were used to construct haplotypes. These were phased using the PHASE program [30] and tested for association using PLINK. The ancestral haplotype was constructed from the chimpanzee (PanTro2) allele whenever possible, and otherwise from the macaque alleles. An ancestral recombination graph was constructed using the program Beagle [13], [31] which allows recombination assuming an infinite site mutation model. After identifying the first recombination event the haplotype segment before the recombination spot was used to construct a median –joining network using the Network program [14]. The alleles of the imputed SNP rs9378212 were then phased on each haplotype segment using the PHASE program.

The URLs for the data and analytic approaches presented herein are as follows:

1000 Genomes http://1000genomes.org

HapMapII http://www.hapmap.org

IMPUTEv1 https://mathgen.stats.ox.ac.uk/impute/impute_v1.html

mRNA by SNP browser http://www.sph.umich.edu/csg/liang/asthma/

R script for recombination plot http://www.broadinstitute.org/science/projects/diabetes-genetics-initiative/plotting-genome-wide-association-results

## Supporting Information

Figure S1 Forest plots of main associations with risk of follicular lymphoma (FL) and diffuse large B-cell lymphoma (DLBCL).
(0.18 MB PDF)

Figure S2 Association results for imputed SNPs and genotyped SNPs.
(0.03 MB PDF)

Figure S3 Ancestral reconstruction graph based on the 14 SNPs in the Stage 1 samples.
(0.05 MB PDF)

Figure S4 Testing of population structure using principal components analysis.
(0.11 MB PDF)

Figure S5 Principal components analysis scree plot.
(0.02 MB PDF)

Figure S6 Quantile-quantile plots before and after genomic control correction.
(0.07 MB PDF)

Table S1 Number of patients with Non-Hodgkin lymphoma subtypes other than follicular lymphoma.
(0.01 MB PDF)

Table S2 Overlap of samples from the current genome-wide association study and the previous GWAS reporting association between 6p21.32 and follicular lymphoma risk.
(0.01 MB PDF)

Table S3 Top 40 SNPs taken forward to Stage 2, sorted by significance level (trend P-value) of association with risk of follicular lymphoma.
(0.02 MB PDF)

Table S4 SNPs on chromosome 6p21.32 that showed genome-wide per allele P-values < 1E-04 in association with risk of follicular lymphoma in Stage 1, sorted by position.
(0.01 MB PDF)

Table S5 Summary statistics for associations with risk of follicular lymphoma in Stages 1 and 2 with combined P-values.
(0.02 MB PDF)

Table S6 Crude and adjusted logistic regression analyses of the six SNPs in 6p21.32 showing significant association with risk of follicular lymphoma in Stages 1 and 2.
(0.01 MB PDF)

Table S7 Individual study results for associations between the 5 SNPs taken forward to Stage 3 and risk of follicular lymphoma in Stage 3.
(0.01 MB PDF)

Table S8 Genotype counts of main SNPs per Cases/Controls, per study and in total.
(0.01 MB PDF)

Table S9 Associations with risk of follicular lymphoma for haplotypes phased with 14 SNPs or 7 SNPs based on genotyped SNPs in Stage 1.
(0.01 MB PDF)

Table S10 Imputation of HLA class II alleles and risk of follicular lymphoma.
(0.01 MB PDF)

Table S11 Trend p-value of associations of age and sex with main genotypes among controls subjects per study.
(0.02 MB PDF)

Text S1 Additional description of validation study subjects.
(0.04 MB PDF)
