# Supplementary material for: GWAS of Follicular Lymphoma Reveals Allelic Heterogeneity at 6p21.32 and Suggests Shared Genetic Susceptibility with Diffuse Large B-cell Lymphoma
Source: PLoS Genet. 2011 Apr 21;7(4):e1001378. doi: 10.1371/journal.pgen.1001378 (PMC3080853; doi:10.1371/journal.pgen.1001378)
Supplement: Figure S1 — Forest plots of main associations with risk of follicular lymphoma (FL) and diffuse large B-cell lymphoma (DLBCL). (0.18 MB PDF) [file pgen.1001378.s001.pdf]

Figure S1. Forest plots of main associations with risk of follicular lymphoma (FL) and diffuse large B-cell lymphoma (DLBCL)

A

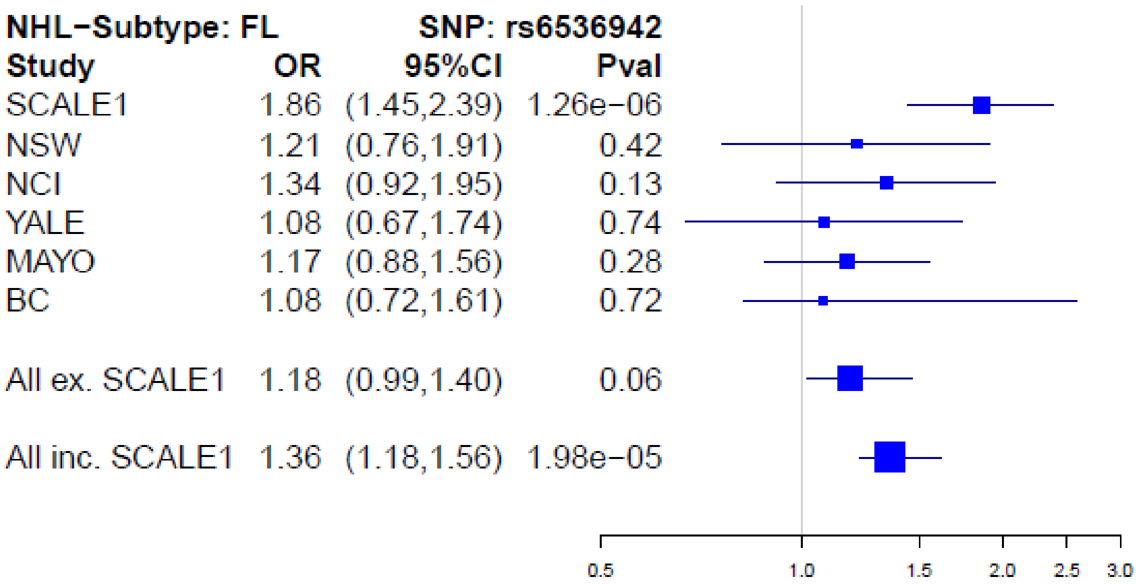

$P_{heterogeneity}$ = 0.09

B

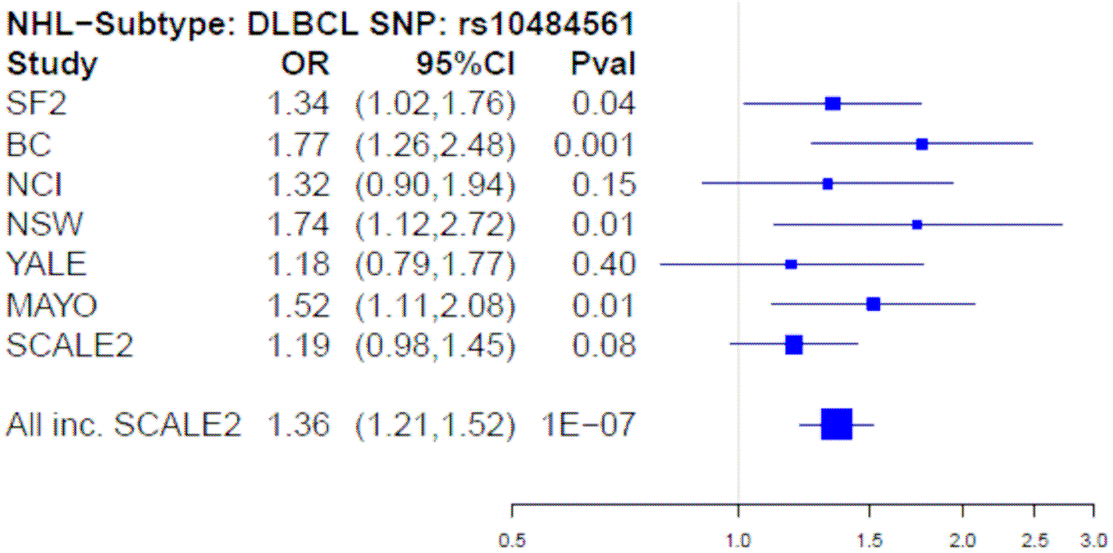

$P_{heterogeneity}$ = 0.28

Abbreviations: SNP: single nucleotide polymorphism, OR: odds ratio, CI: confidence interval, SCALE: Scandinavian lymphoma etiology, SF: San Francisco, BC: British Columbia, NCI: National Cancer Institute-Surveillance, Epidemiology and End Results, NSW: New South Wales, YALE: Yale University, MAYO: Mayo Clinic.
