# Supplementary material for: GWAS of Follicular Lymphoma Reveals Allelic Heterogeneity at 6p21.32 and Suggests Shared Genetic Susceptibility with Diffuse Large B-cell Lymphoma
Source: PLoS Genet. 2011 Apr 21;7(4):e1001378. doi: 10.1371/journal.pgen.1001378 (PMC3080853; doi:10.1371/journal.pgen.1001378)

Figure S2. Association results (trend test on Stage 1, follicular lymphoma) for imputed single nucleotide polymorphism (SNPs) (black) and genotyped SNPs (red). rs9378212 is the top SNP among those showing high (>95%) concordance between HapMap and 1000 Genomes CEU genotypes.

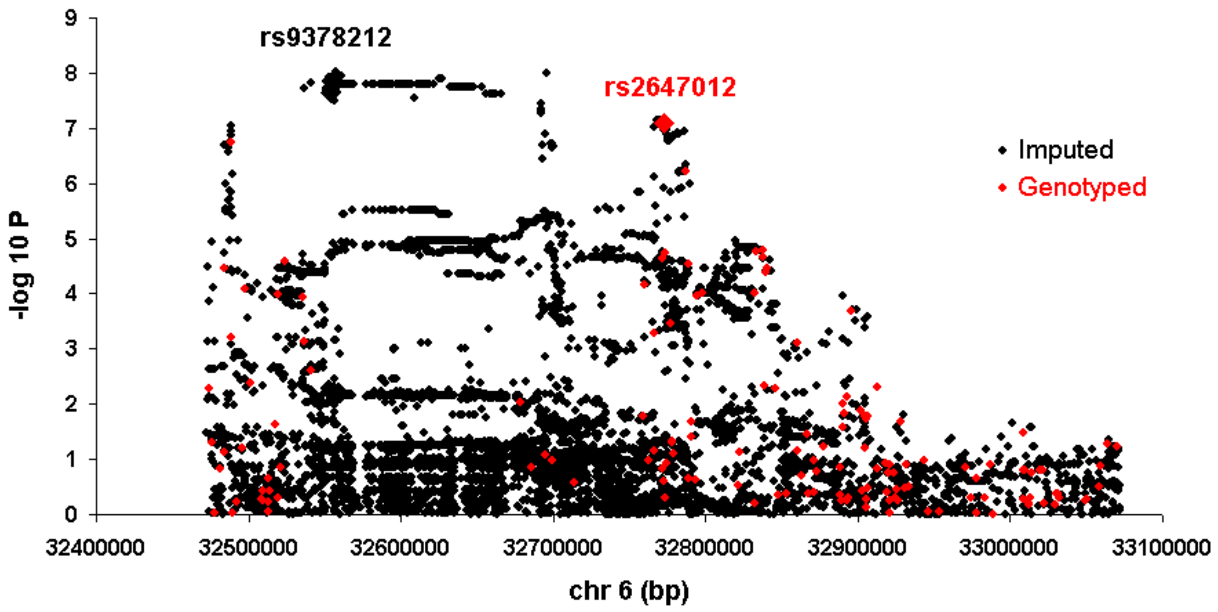

Supplement: Figure S2 — Association results for imputed SNPs and genotyped SNPs. (0.03 MB PDF) [file pgen.1001378.s002.pdf]
