# Supplementary material for: GWAS of Follicular Lymphoma Reveals Allelic Heterogeneity at 6p21.32 and Suggests Shared Genetic Susceptibility with Diffuse Large B-cell Lymphoma
Source: PLoS Genet. 2011 Apr 21;7(4):e1001378. doi: 10.1371/journal.pgen.1001378 (PMC3080853; doi:10.1371/journal.pgen.1001378)

Figure S3. Ancestral reconstruction graph based on the 14 SNPs (Table S9) in the Stage 1 samples (follicular lymphoma). The red boxes are haplotypes, the green ovals are standard coalescent nodes and the blue ones are recombination nodes; the recombination spot is denoted by a number, indicating the base position in the haplotype along with a '-'. The 'L' and 'R' are the left and the right haplotype segments of the recombination spot. SNPs (the associated ones in red) are shown on the black lines.

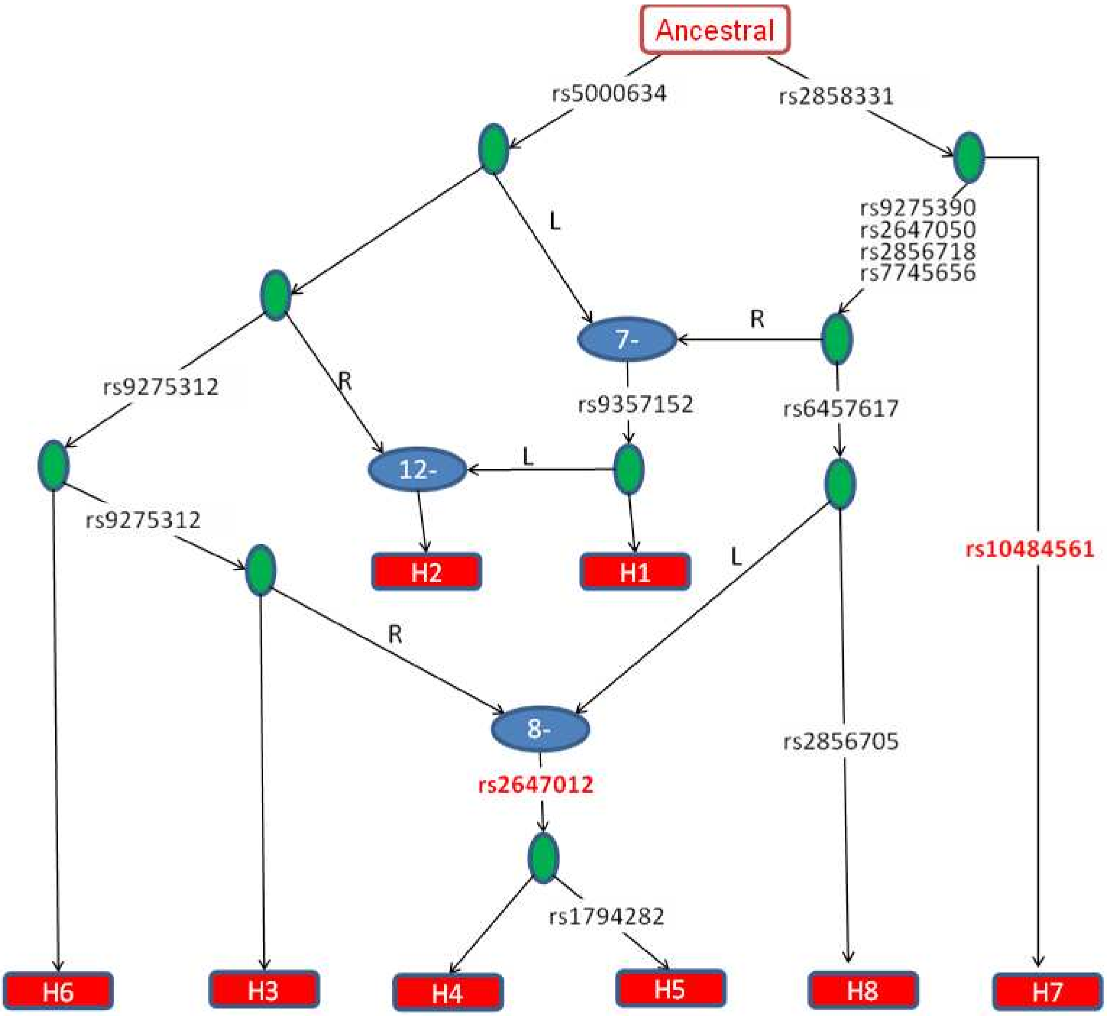

Supplement: Figure S3 — Ancestral reconstruction graph based on the 14 SNPs in the Stage 1 samples. (0.05 MB PDF) [file pgen.1001378.s003.pdf]
