# Supplementary material for: GWAS of Follicular Lymphoma Reveals Allelic Heterogeneity at 6p21.32 and Suggests Shared Genetic Susceptibility with Diffuse Large B-cell Lymphoma
Source: PLoS Genet. 2011 Apr 21;7(4):e1001378. doi: 10.1371/journal.pgen.1001378 (PMC3080853; doi:10.1371/journal.pgen.1001378)

Figure S4. Testing of population structure using principal components analysis (PCA), comparing participants in Swedish (SWE) and Danish (DK) parts of the Scandinavian lymphoma etiology study and Swedish control subjects from the Eira study (EIRA) in the genome-wide association study (Stage 1) cohort on the first three dimensions.

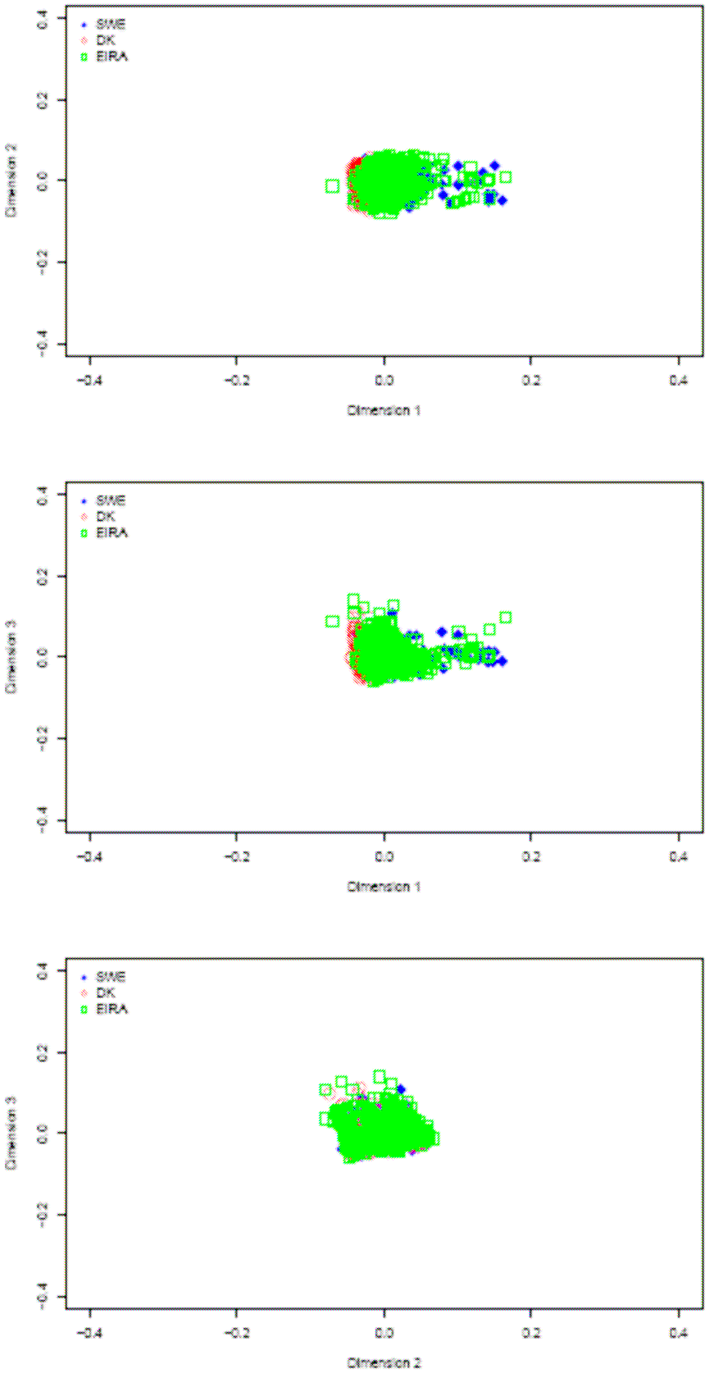

Supplement: Figure S4 — Testing of population structure using principal components analysis. (0.11 MB PDF) [file pgen.1001378.s004.pdf]
