# Supplementary figures and images for: GWAS of Follicular Lymphoma Reveals Allelic Heterogeneity at 6p21.32 and Suggests Shared Genetic Susceptibility with Diffuse Large B-cell Lymphoma
Source: PLoS Genet. 2011 Apr 21;7(4):e1001378. doi: 10.1371/journal.pgen.1001378 (PMC3080853; doi:10.1371/journal.pgen.1001378)

**Figure S5.** Principal components analysis (PCA) scree plot

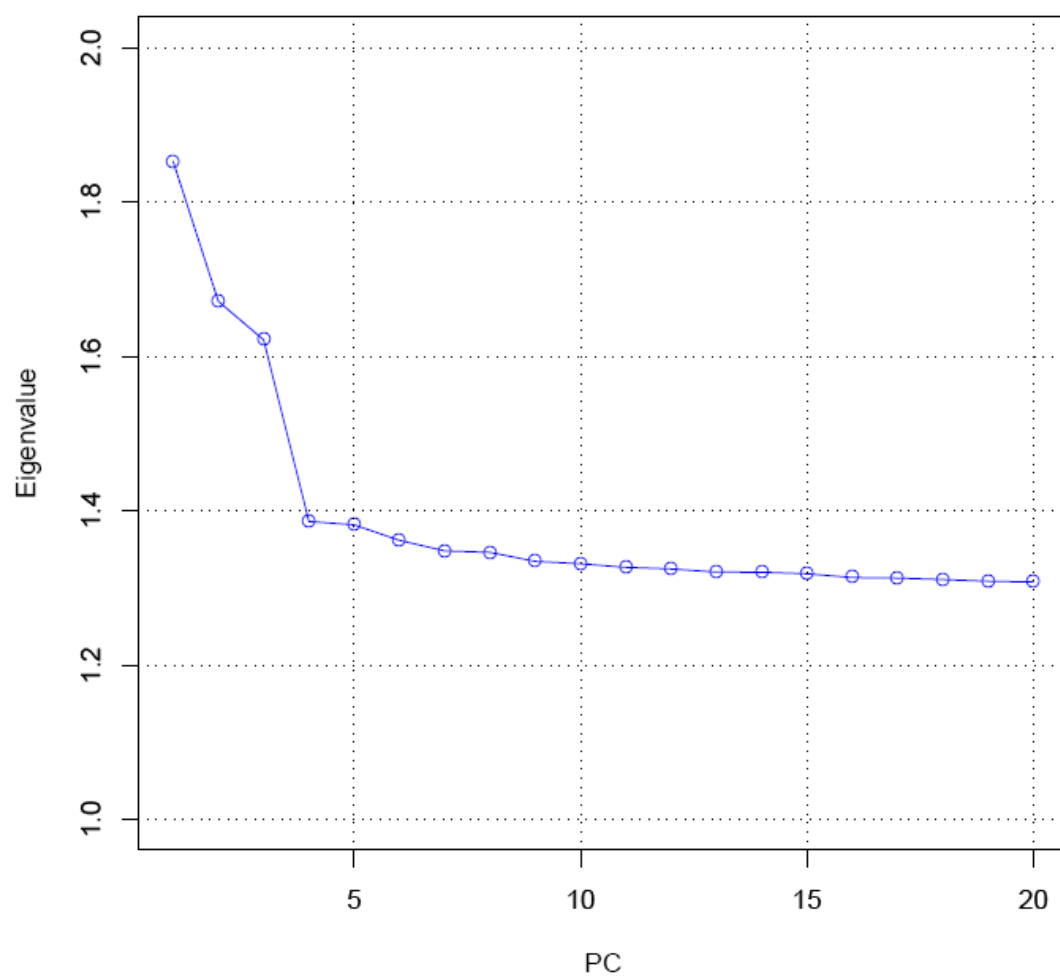

Supplement: Figure S5 — Principal components analysis scree plot. (0.02 MB PDF) [file pgen.1001378.s005.pdf]
