# Supplementary material for: GWAS of Follicular Lymphoma Reveals Allelic Heterogeneity at 6p21.32 and Suggests Shared Genetic Susceptibility with Diffuse Large B-cell Lymphoma
Source: PLoS Genet. 2011 Apr 21;7(4):e1001378. doi: 10.1371/journal.pgen.1001378 (PMC3080853; doi:10.1371/journal.pgen.1001378)
Supplement: Figure S6 — Quantile-quantile plots before and after genomic control correction. (0.07 MB PDF) [file pgen.1001378.s006.pdf]

Figure S6. Quantile-quantile (QQ) plot before (left) and after (right) genomic control correction.

SCALE1 – QQ Plot Crude Analysis<br>Inflation Factor: 1.03941	SCALE1 – QQ Plot PCA Adjusted Analysis<br>Inflation Factor: 1.02829
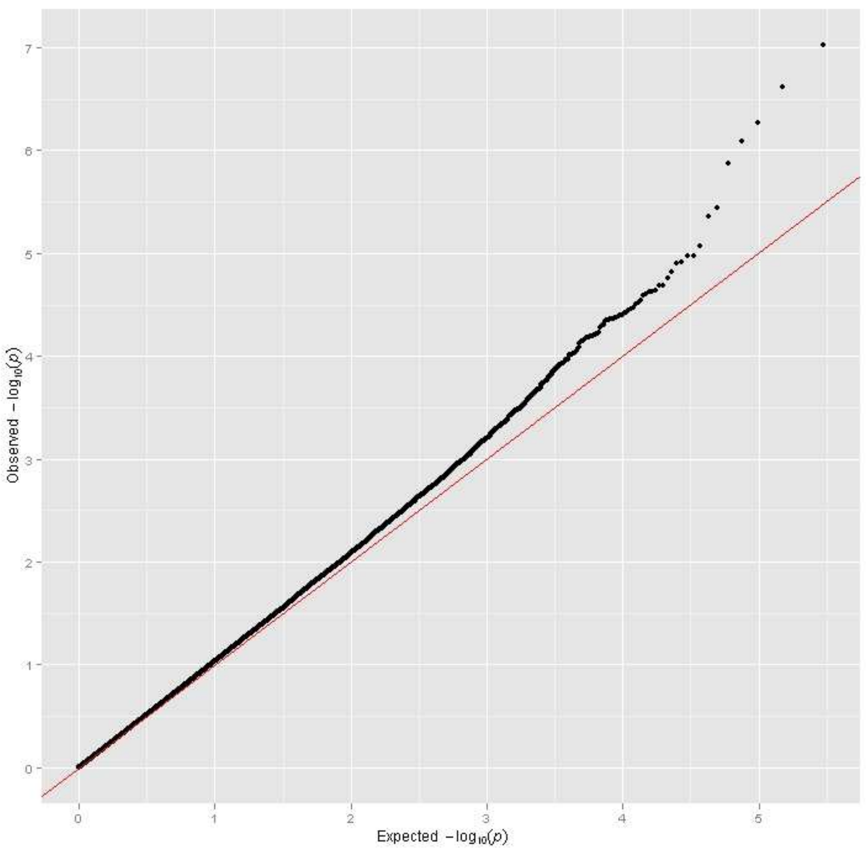	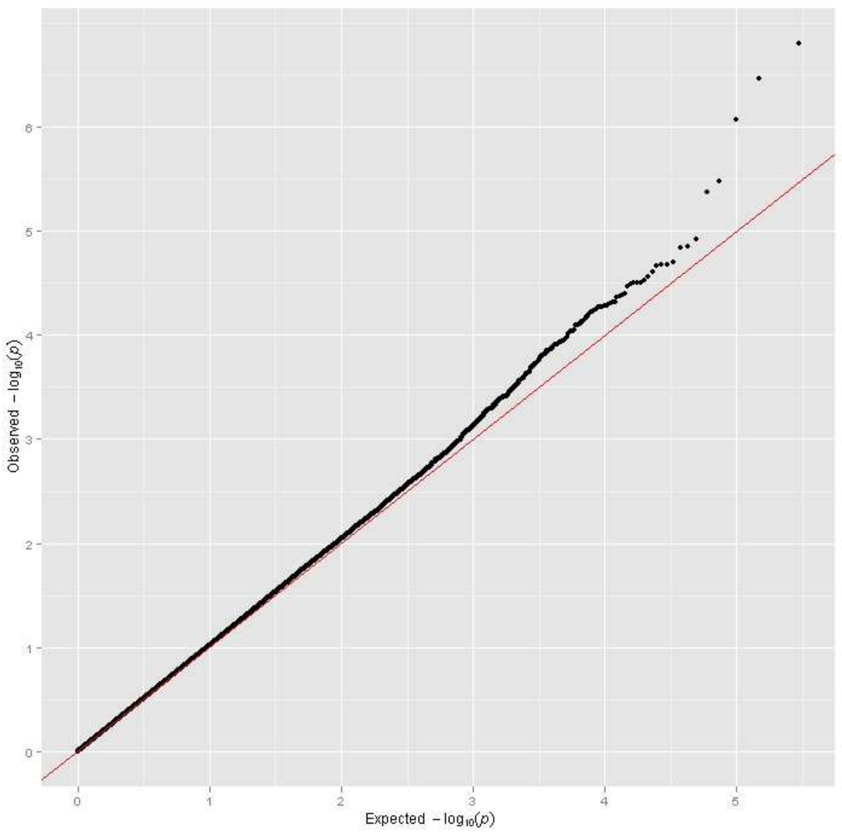

PCA: principal components analysis.
