# Supplementary material for: GWAS of Follicular Lymphoma Reveals Allelic Heterogeneity at 6p21.32 and Suggests Shared Genetic Susceptibility with Diffuse Large B-cell Lymphoma
Source: PLoS Genet. 2011 Apr 21;7(4):e1001378. doi: 10.1371/journal.pgen.1001378 (PMC3080853; doi:10.1371/journal.pgen.1001378)
Supplement: Table S1 — Number of patients with Non-Hodgkin lymphoma subtypes other than follicular lymphoma. (0.01 MB PDF) [file pgen.1001378.s007.pdf]

Table S1. Number of patients with Non-Hodgkin lymphoma (NHL) subtypes other than follicular lymphoma (FL).

Stage	Study	DLBCL [a]	CLL/SLL [b]	Marginal zone lymphoma	Mantle cell lymphoma	T-cell lymphoma	Other rare or unspec NHL [c]
3	NCI-SEER	160	66	51	24	37	130
	NSW	124	15	40	16	9	57
	Yale	125	38	28	10	27	72
	BC	163	37	68	43	50	90
	Mayo	193	327	61	53	34	175
	SCALE2	570	592	88	116	149	354
	SF2	257					
**Total**		**1592**	**1075**	**336**	**262**	**306**	**878**

[a] Diffuse large B-cell lymphoma (DLBCL)

[b] Chronic lymphocytic leukemia/small lymphocytic lymphoma (CLL/SLL)

[c] Includes samples of patients with rare NHL subtypes such as lymphoplasmacytic lymphoma, hairy cell leukemia and prolymphocytic leukemia, unspecified B-cell NHL and unspecified NHL

Abbreviations: SCALE: Scandinavian lymphoma etiology, SF: San Francisco, NCI-SEER: National Cancer Institute, NSW: New South Wales, Yale: Yale University, BC: British Columbia, Mayo: Mayo Clinic.
