# Supplementary material for: GWAS of Follicular Lymphoma Reveals Allelic Heterogeneity at 6p21.32 and Suggests Shared Genetic Susceptibility with Diffuse Large B-cell Lymphoma
Source: PLoS Genet. 2011 Apr 21;7(4):e1001378. doi: 10.1371/journal.pgen.1001378 (PMC3080853; doi:10.1371/journal.pgen.1001378)
Supplement: Table S2 — Overlap of samples from the current genome-wide association study and the previous GWAS reporting association between 6p21.32 and follicular lymphoma risk. (0.01 MB PDF) [file pgen.1001378.s008.pdf]

Table S2. Overlap of samples from the current genome-wide association study (GWAS) and the previous GWAS [1] reporting association between 6p21.32 and follicular lymphoma (FL) risk.

Current GWAS Study			*Previous GWAS Study*[6]			
**Stage**	**Study**	**Samples cases/controls**	**Stage**	**Study**	**Samples cases/controls**	**Comments**
**FL Stage 1**	**SCALE1**	379/791	**FL Stage 1**	**SF1**	213/750	The current and the previous GWAS studies were conducted in two different cohorts (SCALE1 and SF1, respectively), providing different top-associated SNPs that were taken forward to Stage 2[a]
**FL Stage 2**	**SF1**	213/750	**FL Stage 2**	**SCALE**	379/791	The previous GWAS study used 38 SNPs from the current SCALE1 in Stage 2 validation<br>The current GWAS study used 39 SNPs from the previous SF1 in Stage 2 validation[a]
**FL Stage 3**	**NCI-SEER**	157/501	**FL Stage 3**	**NCI-SEER**	153/482	
	**NSW**	161/398		**NSW**	141/369	
	**Yale**	98/460		**Yale**	98/460	
	**BC**	174/610		**BC**	174/610	
	**Mayo**	246/1233		**-**	-	
**FL Stages 1-3**	**Combined**	1428/4743	**FL Stages 1-3**	**Combined**	1158/3462	75% of participants in the current FL GWAS study were included in the previous study
**Evaluation**	**SCALE2**	1869/2018	**DLBCL,**	**-**	-	
**other subtypes**	**NCI-SEER**	468	**CLL/SLL**	**NCI-SEER**	219	
	**NSW**	261	**Stage 3**	**NSW**	118	
	**Yale**	300		**Yale**	38	
	**BC**	451		**BC**	200	
	**Mayo**	843		**-**	-	
	**SF2**	257	**DLBCL Stage 1**	**SF1**	257	
	**Combined**	4449/2018		**Combined**	832	13% of participants in the current evaluation of FL-associated SNPs among other subtypes were included in the previous study
**Grand total**		5877/6761			2090/3462	Overall, 44 % of participants in the current study were included in the previous study

[a]rs10484561 was the only SNP that ranked among the top 40 in both the current and the previous FL GWAS studies, only adjusted results for rs10484561 are presented in the current study. Abbreviations: SCALE: Scandinavian lymphoma etiology, SF: San Francisco, NCI-SEER: National Cancer Institute, NSW: New South Wales, Yale: Yale University, BC: British Columbia, Mayo: Mayo Clinic.
