# Supplementary material for: GWAS of Follicular Lymphoma Reveals Allelic Heterogeneity at 6p21.32 and Suggests Shared Genetic Susceptibility with Diffuse Large B-cell Lymphoma
Source: PLoS Genet. 2011 Apr 21;7(4):e1001378. doi: 10.1371/journal.pgen.1001378 (PMC3080853; doi:10.1371/journal.pgen.1001378)
Supplement: Table S3 — Top 40 SNPs taken forward to Stage 2, sorted by significance level (trend P-value) of association with risk of follicular lymphoma. (0.02 MB PDF) [file pgen.1001378.s009.pdf]

Table S3. Top 40 SNPs taken forward to Stage 2, sorted by significance level (trend *P*-value) of association with risk of follicular lymphoma.

**Chromosomal location**	**SNP**	**Gene**[a]	**Position**	**Minor /major allele**	**MAF controls**	**HWE**	**Per allele trend *P*-value**[b]	**PCA adjusted trend *P*-value**[b]	**PCA adjusted OR (95% CI)**[b]
rs2647012	6p21.32	*HLA-DQB1*	32772436	A/G	0.44	0.71	1.10E-07	1.59E-07	0.58 (0.47,0.71)
rs6932542	6p21.32	*BTNL2*	32488240	A/G	0.50	0.48	2.33E-07	3.43E-07	0.61 (0.50,0.73)
rs9275572	6p21.32	*HLA-DQA2*	32786977	A/G	0.48	0.81	7.30E-07	8.43E-07	0.61 (0.50,0.74)
rs6536942	4q32.3	*TLL1*	167205644	G/A	0.12	0.18	1.14E-06	4.23E-06	1.81 (1.41,2.33)
rs716183	10q25.3	*VAX1*	118894485	C/T	0.36	0.20	1.59E-06	3.31E-06	1.52 (1.27,1.82)
rs504985	6q25.1	*TAB2*	149700671	G/T	0.36	0.76	3.80E-06	2.17E-05	1.48 (1.24,1.78)
rs11819305	10q25.3	*VAX1*	118898040	A/G	0.31	0.02	1.03E-05	1.97E-05	1.48 (1.23,1.76)
rs1912822	4q32.3	*MARCH1*	164744059	A/G	0.34	0.57	1.03E-05	1.44E-05	1.50 (1.25,1.80)
rs1860373	17q23.2	*BCAS3*	56643678	C/T	0.16	0.77	1.16E-05	3.14E-05	1.60 (1.28,1.99)
rs2868145	19q13.3	*PDCD5*	37738954	G/A	0.14	0.67	1.24E-05	3.20E-05	1.63 (1.29,2.05)
rs2179367	6q25.1	*ZC3H12D*	149804230	C/T	0.37	1.00	1.37E-05	7.10E-05	1.44 (1.20,1.72)
rs7453920	6p21.32	*HLA-DQB2*	32837990	A/G	0.50	0.005	1.83E-05	5.31E-05	0.67 (0.56,0.82)
rs2301271	6p21.32	*HLA-DQB2*	32833171	T/C	0.50	0.004	1.91E-05	5.59E-05	0.67 (0.56,0.82)
rs3817973	6p21.32	*BTNL2*	32469089	A/G	0.42	0.48	1.94E-05	5.41E-05	1.48 (1.22,1.79)
rs9498061	6q24.3	*SASH1*	148900981	G/A	0.23	0.53	2.04E-05	2.11E-05	0.60 (0.47,0.76)
rs4424066	6p21.32	*BTNL2*	32462406	G/A	0.42	0.48	2.21E-05	6.04E-05	1.48 (1.22,1.78)
rs2457058	1p33	-	47875279	C/T	0.27	0.58	2.31E-05	3.39E-05	1.50 (1.24,1.82)
rs10484561	6p21.32	*HLA-DQB1*	32773398	G/T	0.11	0.20	2.33E-05	5.77E-05	1.67 (1.30,2.14)
rs2051549	6p21.32	*HLA-DQB2*	32838064	C/T	0.50	0.007	2.42E-05	6.92E-05	0.68 (0.56,0.82)
rs6457617	6p21.32	*HLA-DQB1*	32771829	C/T	0.51	0.95	2.64E-05	2.94E-05	0.66 (0.55,0.80)
rs13209234	6p21.32	*HLA-DRA*	32523953	A/G	0.14	0.75	3.10E-05	4.14E-05	1.64 (1.29,2.07)
rs881633	16q24.3	*CTU2*	87301239	A/G	0.08	0.51	3.13E-05	2.10E-05	1.85 (1.39,2.46)
rs4447756	3p14.2	*PTPRG*	61939996	C/A	0.44	0.55	3.23E-05	1.42E-05	0.66 (0.55,0.80)
rs2076530	6p21.32	*BTNL2*	32471794	G/A	0.43	0.56	3.28E-05	7.74E-05	1.47 (1.21,1.77)
rs2858331	6p21.32	*HLA-DQA2*	32789255	C/T	0.30	0.14	3.30E-05	1.09E-04	1.46 (1.21,1.78)
rs934320	10q26.12	*WDR11*	122694003	T/C	0.47	0.51	3.34E-05	4.85E-05	0.69 (0.58,0.82)
rs6477205	9p24.1	-	7506770	T/G	0.35	0.42	3.67E-05	1.12E-04	1.44 (1.2,1.72)

rs1015735	18q12.3	-	39877321	C/T	0.26	0.56	3.84E-05	9.68E-05	1.48 (1.21,1.80)
rs4340393	18q12.3	-	39884052	T/C	0.25	0.56	4.03E-05	9.05E-05	1.48 (1.22,1.80)
rs1386706	13q31.1	-	82314548	A/G	0.04	0.13	4.06E-05	3.13E-05	2.17 (1.51,3.13)
rs1386706	13q31.1	-	82299724	G/A	0.04	0.002	4.06E-05	3.13E-05	2.17 (1.51,3.13)
rs6029356	20q12	-	38882719	A/G	0.15	0.69	4.18E-05	3.15E-05	0.53 (0.39,0.71)
rs9498335	6q25.1	*TAB2*	149735477	A/C	0.26	0.61	4.18E-05	1.23E-04	1.47 (1.21,1.78)
rs10214840	6q25.1	*TAB2*	149685402	C/T	0.26	0.66	4.35E-05	1.27E-04	1.46 (1.21,1.78)
rs7613835	3q26.31	*NAALADL2*	176632062	G/A	0.26	0.93	4.40E-05	2.77E-05	0.63 (0.50,0.78)
rs9277554	6p21.32	*HLA-DPB1*	33163516	T/C	0.28	0.08	4.53E-05	1.12E-04	0.66 (0.54,0.82)
rs6793266	3q26.31	*NAALADL2*	176585032	T/C	0.43	0.27	6.30E-05	1.23E-04	0.70 (0.58,0.84)
rs4891489	18q22.3	*NETO1*	68662302	A/G	0.40	0.12	6.59E-05	2.43E-04	0.71 (0.59,0.85)
rs441890	8q13.3	*LACTB2*	71727221	G/A	0.40	0.11	6.80E-05	1.19E-04	1.41 (1.18,1.68)
rs1552126	14q32.2	-	97063843	C/T	0.42	0.02	8.46E-05	1.86E-04	0.71 (0.60,0.85)

[a] Closest gene located within 50 kb distance of the marker

[b] Per allele trend *P*-value, Odds ratio (OR) and 95% confidence interval (CI) adjusted for principal components analysis (PCA)

MAF: minor allele frequency, HWE: Hardy-Weinberg equilibrium
