# Supplementary material for: GWAS of Follicular Lymphoma Reveals Allelic Heterogeneity at 6p21.32 and Suggests Shared Genetic Susceptibility with Diffuse Large B-cell Lymphoma
Source: PLoS Genet. 2011 Apr 21;7(4):e1001378. doi: 10.1371/journal.pgen.1001378 (PMC3080853; doi:10.1371/journal.pgen.1001378)
Supplement: Table S4 — SNPs on chromosome 6p21.32 that showed genome-wide per allele P-values < 1E-04 in association with risk of follicular lymphoma in Stage 1, sorted by position. (0.01 MB PDF) [file pgen.1001378.s010.pdf]

Table S4. SNPs on chromosome 6p21.32 that showed genome-wide per allele *P*-values < 10^{-4} in association with risk of follicular lymphoma in Stage 1, sorted by position.

**Chromosomal location**	**SNP**	**Gene**[a]	**Position**	**MAF controls**	**HWE controls**	**Per allele trend *P*-value**	**PCA adjusted trend *P*-value** [b]	**PCA adjusted OR (95% CI)** [b]
rs4424066	6p21.32	BTNL2 (8)	32462406	0.42	0.66	2.21E-05	6.04E-05	1.48 (1.22-1.78)
rs3817973	6p21.32	BTNL2 (1.4)	32469089	0.42	0.72	1.94E-05	5.41E-05	1.48 (1.22-1.79)
rs2076530	6p21.32	BTNL2 (missense)	32471794	0.43	0.72	3.28E-05	7.74E-05	1.47 (1.21-1.77)
rs3763313	6p21.32	BTNL2 (-1.5)	32484449	0.19	0.17	4.00E-05	7.30E-05	1.55 (1.25-1.92)
rs6932542	6p21.32	BTNL2 (-5)	32488240	0.50	0.39	2.33E-07	3.43E-07	0.61 (0.50-0.73)
rs3135363	6p21.32	HLA-DRA (-15)	32497626	0.30	0.18	9.10E-05	7.94E-05	0.62 (0.49-0.79)
rs13209234	6p21.32	HLA-DRA (3)	32523953	0.14	0.31	3.10E-05	4.14E-05	1.64 (1.29-2.07)
rs7755224	6p21.32	HLA-DQB1 (-17)	32760295	0.11	0.86	8.11E-05	1.39E-04	1.63 (1.27-2.10)
rs6457617	6p21.32	HLA-DQB1 (-29)	32771829	0.51	0.57	2.64E-05	2.94E-05	0.66 (0.55-0.80)
rs2647012	6p21.32	HLA-DQB1(-29)	32772436	0.44	0.47	1.10E-07	1.59E-07	0.58 (0.47-0.71)
rs10484561	6p21.32	HLA-DQB1 (-30)	32773398	0.11	0.86	2.33E-05	5.77E-05	1.67 (1.30-2.14)
rs9275572	6p21.32	HLA-DQA2 (-30)	32786977	0.48	1.00	7.30E-07	8.43E-07	0.61 (0.50-0.74)
rs2858331	6p21.32	HLA-DQA2 (-27)	32789255	0.30	0.21	3.30E-05	1.09E-04	1.46 (1.21-1.78)
rs2301271	6p21.32	HLA-DQB2 (intron)	32833171	0.50	0.01	1.91E-05	5.59E-05	0.67 (0.56-0.82)
rs7453920	6p21.32	HLA-DQB2 (intron)	32837990	0.50	0.01	1.83E-05	5.31E-05	0.67 (0.56-0.82)
rs2051549	6p21.32	HLA-DQB2 (intron)	32838064	0.50	0.01	2.42E-05	6.92E-05	0.68 (0.56-0.82)
rs1573649	6p21.32	HLA-DQB2 (5' UTR)	32839236	0.43	0.04	4.46E-05	9.09E-05	1.46 (1.21-1.76)
rs6903130	6p21.32	HLA-DQB2 (-0.8)	32840188	0.43	0.03	3.93E-05	6.65E-05	1.48 (1.22-1.79)
rs9277554	6p21.32	HLA-DPB1(0.6)	33163516	0.28	0.10	4.53E-05	1.12E-04	0.66 (0.54-0.82)

[a] Closest gene located within 50 kb of the marker, shown with distance of SNP upstream (-) or downstream (+) of gene.

[b] Per allele trend *P*-value, Odds ratio (OR) and 95% confidence interval (CI) adjusted for principal components analysis (PCA)

MAF: minor allele frequency, HWE: Hardy-Weinberg equilibrium
