# Supplementary material for: GWAS of Follicular Lymphoma Reveals Allelic Heterogeneity at 6p21.32 and Suggests Shared Genetic Susceptibility with Diffuse Large B-cell Lymphoma
Source: PLoS Genet. 2011 Apr 21;7(4):e1001378. doi: 10.1371/journal.pgen.1001378 (PMC3080853; doi:10.1371/journal.pgen.1001378)
Supplement: Table S5 — Summary statistics for associations with risk of follicular lymphoma in Stages 1 and 2 with combined P-values. (0.02 MB PDF) [file pgen.1001378.s011.pdf]

Table S5. Summary statistics for associations[a] with risk of follicular lymphoma in Stages 1 and 2 with combined *P*-values. SNPs in bold were taken forward to Stage 3. The closest gene within 50Kb is shown.

Chromosomal location	SNP	Gene	Position	Minor /major allele	SCALE1			SF			Combined	
					MAF Controls	OR(95% CI)	P-value	MAF Controls	OR (95% CI)	P-value	OR (95% CI)	P-value
**rs2647012**	**6p21.32**	***HLA-DQB1***	**32772436**	**A/G**	**0.44**	**0.61 (0.51-0.73)**	**1.10E-07**	**0.36**	**0.64 (0.47-0.86)**	**4.77E-03** [b]	**0.62 (0.53-0.72)**	**1.64E-09**
rs6932542	6p21.32	*BTNL2*	32488240	A/G	0.5	0.63 (0.53-0.75)	2.33E-07	0.48	0.64 (0.46-0.88)	1.70E-02 [b]	0.63 (0.54-0.74)	5.30E-09
rs9275572	6p21.32	*HLA-DQA2*	32786977	A/G	0.48	0.64 (0.53-0.76)	7.30E-07	0.39	0.59 (0.44-0.81)	5.52E-04 [b]	0.62 (0.54-0.73)	2.09E-09
**rs6536942**	**4q32.3**	***TLL1***	**167205644**	**G/A**	**0.12**	**1.80 (1.42-2.29)**	**1.14E-06**	-	-	-	-	-
**rs716183**	**10q25.3**	***VAX1***	**118894485**	**C/T**	**0.36**	**1.56 (1.30-1.86)**	**1.59E-06**	**0.42**	**1.16 (0.84-1.62)**	**0.42**	**1.46 (1.25.1.70)**	**1.88E-06**
rs504985	6q25.1	*TAB2*	149700671	G/T	0.36	1.52 (1.27-1.81)	3.80E-06	0.4	1.12 (0.81-1.56)	0.47	1.42 (1.21-1.65)	1.10E-05
rs11819305	10q25.3	*VAX1*	118898040	A/G	0.31	1.52 (1.27-1.83)	1.03E-05	0.36	1.04 (0.76-1.42)	0.8	1.38 (1.18-1.62)	4.68E-05
rs1912822	4q32.3	*MARCH1*	164744059	A/G	0.34	1.49 (1.25-1.78)	1.03E-05	0.36	1.18 (0.86-1.62)	0.44	1.41 (1.21-1.64)	1.44E-05
rs1860373	17q23.2	*BCAS3*	56643678	C/T	0.16	1.61 (1.30-2.00)	1.16E-05	0.15	1.00 (0.71-1.41)	0.94	1.41 (1.18-1.69)	2.00E-04
rs2868145	19q13.3	*PDCD5*	37738954	G/A	0.14	1.66 (1.33-2.08)	1.24E-05	0.15	1.24 (0.89-1.72)	0.29	1.51 (1.26-1.82)	1.21E-05
rs2179367	6q25.1	*ZC3H12D*	149804230	C/T	0.37	1.48 (1.24-1.76)	1.37E-05	0.41	1.15 (0.83-1.60)	0.42	1.40 (1.20-1.63)	2.24E-05
rs7453920	6p21.32	*HLA-DQB2*	32837990	A/G	0.5	0.69 (0.58-0.82)	1.83E-05	0.41	0.72 (0.53-0.99)	5.90E-02	0.70 (0.60-0.81)	4.40E-06
rs2301271	6p21.32	*HLA-DQB2*	32833171	T/C	0.5	0.69 (0.58-0.82)	1.91E-05	0.41	0.72 (0.53-0.99)	5.50E-02	0.70 (0.60-0.82)	4.21E-06
rs3817973	6p21.32	*BTNL2*	32469089	A/G	0.42	1.46 (1.22-1.74)	1.94E-05	0.42	1.08 (0.78-1.50)	0.12	1.36 (1.17-1.59)	7.30E-05
rs4424066	6p21.32	*BTNL2*	32462406	G/A	0.42	1.46 (1.22-1.73)	2.21E-05	0.42	1.08 (0.78-1.51)	0.12	1.36 (1.17-1.59)	7.67E-05
rs10484561	6p21.32	*HLA-DQB1*	32773398	G/T	0.11	1.72 (1.34-2.19)	2.33E-05	0.13	2.06 (1.48-2.85)	2.67E-05 [b]	1.83 (1.51-2.23)	1.25E-09
rs2051549	6p21.32	*HLA-DQB2*	32838064	C/T	0.5	0.70 (0.58-0.83)	2.42E-05	0.41	0.74 (0.54-1.02)	7.50E-02	0.71 (0.61-0.82)	8.30E-06
rs6457617	6p21.32	*HLA-DQB1*	32771829	C/T	0.51	0.68 (0.58-0.82)	2.64E-05	0.49	0.60 (0.44-0.83)	1.28E-03 [b]	0.66 (0.57-0.78)	2.02E-07
rs13209234	6p21.32	*HLA-DRA*	32523953	A/G	0.14	1.62 (1.29-2.03)	3.10E-05	-	-	-	-	-
rs4447756	3p14.2	*PTPRG*	61939996	C/A	0.44	0.68 (0.57-0.82)	3.23E-05	0.4	0.94 (0.68-1.29)	0.55	0.74 (0.63-0.86)	1.50E-04
rs2076530	6p21.32	*BTNL2*	32471794	G/A	0.43	1.44 (1.21-1.72)	3.28E-05	0.42	1.13 (0.81-1.58)	9.60E-02	1.37 (1.17-1.60)	6.68E-05
rs2858331	6p21.32	*HLA-DQA2*	32789255	C/T	0.3	1.46 (1.22-1.75)	3.30E-05	0.42	1.60 (1.14-2.28)	2.00E-04 [b]	1.49 (1.27-1.75)	1.27E-06
rs934320	10q26.12	*WDR11*	122694003	T/C	0.47	0.68 (0.57-0.82)	3.34E-05	0.45	0.93 (0.67-1.30)	0.89	0.73 (0.63-0.86)	9.43E-05
rs6477205	9p24.1	-	7506770	T/G	0.35	1.45 (1.21-1.73)	3.67E-05	0.35	1.17 (0.86-1.60)	0.92	1.38 (1.18-1.60)	5.13E-05
rs1015735	18q12.3	-	39877321	C/T	0.26	1.48 (1.23-1.80)	3.84E-05	0.31	1.00 (0.74-1.37)	0.99	1.34 (1.14-1.57)	4.50E-04

rs4340393	18q12.3	-	39884052	T/C	0.25	1.48 (1.23-1.79)	4.03E-05	0.31	1.00 (0.74-1.36)	0.95	1.33 (1.13-1.56)	5.10E-04
rs1386706	13q31.1	-	82314548	A/G	0.04	2.21 (1.53-3.19)	4.06E-05	0.06	1.17 (0.74-1.81)	0.34	1.71 (1.29-2.27)	2.10E-04
rs9498335	6q25.1	*TAB2*	149735477	A/C	0.26	1.48 (1.23-1.79)	4.18E-05	0.29	0.99 (0.73-1.34)	0.72	1.32 (1.13.1.56)	5.80E-04
rs10214840	6q25.1	*TAB2*	149685402	C/T	0.26	1.48 (1.22-1.79)	4.35E-05	0.29	0.99 (0.73-1.35)	0.71	1.32 (1.13-1.56)	5.78E-04
**rs9277554**	**6p21.32**	***HLA-DPB1***	**33163516**	**T/C**	**0.28**	**0.64 (0.52-0.79)**	**4.53E-05**	**0.29**	**0.76 (0.55-1.03)**	**9.79E-02**	**0.67 (0.57-0.80)**	**7.39E-06**
**rs441890**	**8q13.3**	***LACTB2***	**71727221**	**G/A**	**0.4**	**1.44 (1.21-1.72)**	**6.80E-05**	**0.42**	**1.42 (1.02-2.02)**	**4.71E-03** [b]	**1.44 (1.23-1.68)**	**4.91E-06**
rs4567602	13q31.1	-	8299724	A/G	0.05	2.21 (1.52-3.19)	7.33E-05	0.06	1.15 (0.73-1.77)	0.39	1.69 (1.27-2.25)	2.90E-04
rs1552126	14q32.2	-	97063843	C/T	0.42	0.69 (0.57-0.82)	8.46E-05	0.43	0.90 (0.65-1.24)	0.81	0.73 (0.62-0.86)	1.13E-04

[a] Per allele odds ratios (OR) with 95% confidence intervals (CI) and per allele *P*-values.

[b] SNPs with *P*-values < 0.05 in association with risk of follicular lymphoma in Stage 2 (SF1 study).

MAF: minor allele frequency, SCALE: Scandinavian lymphoma etiology, SF: San Francisco
