# Supplementary material for: GWAS of Follicular Lymphoma Reveals Allelic Heterogeneity at 6p21.32 and Suggests Shared Genetic Susceptibility with Diffuse Large B-cell Lymphoma
Source: PLoS Genet. 2011 Apr 21;7(4):e1001378. doi: 10.1371/journal.pgen.1001378 (PMC3080853; doi:10.1371/journal.pgen.1001378)
Supplement: Table S6 — Crude and adjusted logistic regression analyses of the six SNPs in 6p21.32 showing significant association with risk of follicular lymphoma in Stages 1 and 2. (0.01 MB PDF) [file pgen.1001378.s012.pdf]

Table S6. Crude and adjusted logistic regression analyses, with odds ratios (ORs) and trend (per allele), P-values, of the six SNPs in 6p21.32 showing significant association with risk of follicular lymphoma in Stages 1 and 2, including rs10484561

**CHR**	**SNP**	**BP**	**Allele**	**Logistic regression, crude**		**Adjusted for rs2647012**		**Adjusted for rs10484561**		**Adjusted for rs2647012 and rs10484561**	
				**OR**	**P**	**OR**	**P**	**OR**	**P**	**OR**	**P**
6	rs6932542	32488240	A	0.62	2.33E-07	0.76	0.04	0.66	2.51E-05	0.79	0.09
6	rs6457617	32771829	C	0.68	2.64E-05	1.09	0.58	0.75	0.003	1.22	0.23
6	rs2647012	32772436	T	0.60	1.10E-07	NA	NA	0.65	1.16E-05	NA	NA
6	rs10484561	32773398	G	1.69	2.33E-05	1.43	0.006	NA	NA	NA	NA
6	rs9275572	32786977	A	0.63	7.30E-07	0.93	0.72	0.68	7.84E-05	1.00	0.99
6	rs2858331	32789255	G	1.49	3.30E-05	1.19	0.12	1.30	0.02	1.03	0.84

CHR: chromosome, BP: base pair
