# Supplementary material for: GWAS of Follicular Lymphoma Reveals Allelic Heterogeneity at 6p21.32 and Suggests Shared Genetic Susceptibility with Diffuse Large B-cell Lymphoma
Source: PLoS Genet. 2011 Apr 21;7(4):e1001378. doi: 10.1371/journal.pgen.1001378 (PMC3080853; doi:10.1371/journal.pgen.1001378)
Supplement: Table S7 — Individual study results for associations between the 5 SNPs taken forward to Stage 3 and risk of follicular lymphoma in Stage 3. (0.01 MB PDF) [file pgen.1001378.s013.pdf]

Table S7. Individual study results for associations between the 5 SNPs taken forward to Stage 3 and risk of follicular lymphoma in Stage 3.

	BC		NCI-SEER		NSW		YALE		Mayo		Joint	
**SNP**	**OR (95%CI)**	**P-value**	**OR (95%CI)**	**P-value**	**OR (95%CI)**	**P-value**	**OR (95%CI)**	**P-value**	**OR (95%CI)**	**P-value**	**OR (95%CI)**	**P-value**
rs2647012 chr6:32772436 *HLA-DQB1*	0.69 (0.53-0.89)	3.84E-03	0.81 (0.61- 1.06)	0.12	0.53 (0.38- 0.73)	1.37E-04	0.63 (0.45- 0.89)	8.58E-03	0.56 (0.45-0.69)	1.15E-07	0.65 (0.58-0.72)	2.69E-15
rs6536942 chr4:167205644 *TLL1*	1.08 (0.72- 1.61)	0.72	1.34 (0.92-1.95)	0.13	1.21 (0.76-1.91)	0.42	1.08 (0.67-1.75)	0.74	1.17 (0.88-1.56)	0.28	1.18 (0.99-1.40)	0.06
rs9275574 chr6:33163516 *HLA-DPB1*	1.05 (0.89-1.36)	0.72	1.02 (0.78-1.33)	0.91	0.79 (0.57-1.10)	0.17	0.84 (0.59-1.19)	0.33	0.91 (0.73-1.13)	0.39	0.91 (0.81-1.01)	0.08
rs441890 chr8:71727221 *LACTB2*	1.12 (0.88- 1.43)	0.35	1.22 (0.93-1.59)	0.14	1.06 (0.78-1.43)	0.71	1.00 (0.73-1.38)	0.97	0.83 (0.68-1.01)	0.06	1.01 (0.90-1.13)	0.91
rs716183 chr10:118894485 *VAX1*	0.87 (0.69-1.11)	0.28	0.99 (0.77-1.27)	0.95	1.34 (1.01-1.79)	0.04	1.03 (0.75- 1.41)	0.85	0.91 (0.74- 1.11)	0.34	0.99 (0.88-1.10)	0.84

SCALE: Scandinavian lymphoma etiology, SF: San Francisco, BC: British Columbia, NCI-SEER: National Cancer Institute-Surveillance, Epidemiology and End Results, NSW: New South Wales, Yale: Yale University, Mayo: Mayo Clinic, OR: odds ratio, CI: confidence interval
