# Supplementary material for: GWAS of Follicular Lymphoma Reveals Allelic Heterogeneity at 6p21.32 and Suggests Shared Genetic Susceptibility with Diffuse Large B-cell Lymphoma
Source: PLoS Genet. 2011 Apr 21;7(4):e1001378. doi: 10.1371/journal.pgen.1001378 (PMC3080853; doi:10.1371/journal.pgen.1001378)
Supplement: Table S8 — Genotype counts of main SNPs per Cases/Controls, per study and in total. (0.01 MB PDF) [file pgen.1001378.s014.pdf]

Table S8. Genotype counts of main single nucleotide polymorphisms (SNPs) per Cases/Controls (Ca/Co); per study and in total.

NHL subtype	SNP	Allel	SCALE1	SF	BC	NCI-SEER	NSW	Mayo	Yale	Total
			Ca/Co	Ca/Co	Ca/Co	Ca/Co	Ca/Co	Ca/Co	Ca/Co	Ca/Co
**FL**	**rs2647012**	GG	173/245	110/304	83/225	79/196	67/100	116/403	55/182	683/1655
	chr6:32772436	GA	166/399	81/342	72/271	52/222	48/155	111/605	32/209	562/2203
	HLA-DQB1	AA	38/145	20/97	16/97	21/61	11/50	17/224	10/64	133/738
**FL**	**rs6536942**	AA	231/605	-	138/489	114/381	96/250	187/980	78/363	844/3068
	chr4:167205644	AG	121/166	-	34/112	33/92	30/49	55/233	17/90	290/742
	TLL1	GG	9/8	-	1/3	5/6	0/6	4/20	3/5	22/48
**FL**	**rs9277554**	CC	241/414	124/381	88/315	80/254	65/135	137/635	55/232	790/2366
	chr6:33163516	CT	121/302	73/297	70/225	55/173	51/139	86/490	36/189	492/1815
	HLA-DPB1	TT	16/73	16/65	15/53	17/52	10/31	21/100	7/39	102/413
**FL**	**rs441890**	AA	108/289	56/252	52/215	48/183	41/108	96/413	31/158	376/1366
	chr8:71727221	AG	168/374	103/365	93/283	78/227	65/148	115/595	51/221	570/1851
	LACTB2	GG	98/126	54/130	28/99	26/69	20/49	35/221	15/81	222/645
**FL**	**rs716183**	TT	115/328	67/261	63/201	53/159	41/101	84/389	28/146	384/1324
	chr10:118894485	TC	175/360	105/352	82/285	65/219	45/153	122/618	53/224	542/1859
	VAX1	CC	88/103	41/137	27/115	34/101	40/51	40//225	17/88	246/683
**DLBCL**	**rs10484561**	TT	-	25/23	113/474	99/343	73/299	144/1005	90/352	944/4016
	chr6:32773398	TG	-	19/23	37/109	42/107	30/66	43/214	29/94	306/987
	HLA-DQB1	GG	-	8/13	9/6	2/5	2/4	6/13	3/9	36/67

FL: follicular lymphoma, DLBCL: diffuse large B-cell lymphoma, SCALE: Scandinavian lymphoma etiology, SF: San Francisco, BC: British Columbia, NCI-SEER: National Cancer Institute-Surveillance, Epidemiology and End Results, NSW: New South Wales, Yale: Yale University, Mayo: Mayo Clinic.
