# Supplementary material for: GWAS of Follicular Lymphoma Reveals Allelic Heterogeneity at 6p21.32 and Suggests Shared Genetic Susceptibility with Diffuse Large B-cell Lymphoma
Source: PLoS Genet. 2011 Apr 21;7(4):e1001378. doi: 10.1371/journal.pgen.1001378 (PMC3080853; doi:10.1371/journal.pgen.1001378)
Supplement: Table S9 — Associations with risk of follicular lymphoma for haplotypes phased with 14 SNPs or 7 SNPs based on genotyped SNPs in Stage 1. (0.01 MB PDF) [file pgen.1001378.s015.pdf]

Table S9. Associations with risk of follicular lymphoma for haplotypes phased with 14 SNPs or 7 SNPs based on genotyped SNPs in Stage 1

Haplotypes (minor allele frequency>1%) were phased[2] with A) 14 SNPs rs5000634, rs6457617, rs2647012, rs9357152, rs10484561, rs9275312, rs1794282, rs9275390, rs2647050, rs2856718, rs2856705, rs9275572, rs7745656, rs2858331 or B) 7 SNPs (rs5000634, rs6457617, rs2647012, rs9357152, rs10484561, rs9275312, rs1794282). Each haplotype was tested for association using a logistic regression test. The derived alleles for rs2647012 and rs10484561 are indicated respectively, in blue and red. SNPs are located from chr6:32771542 (51.26 cM) to 32789255 (51.29 cM), between *HLA-DQB1* and *HLA-DQA2*.

A) 14 SNPs with recombination

Hap	HAPLOTYPE	Freq cases	Freq controls	OR (95% CI)	P
H1	GTCGTACTCTCGTG	0.119	0.118	1.01 (0.77-1.31)	0.97
H2	GTCGTACTCTCGGA	0.092	0.077	1.22 (0.89-1.65)	0.21
H3	GTCATGCCTCCGGA	0.148	0.137	1.09 (0.86-1.40)	0.47
H4	AC**T**ATACTTCCAGA	0.210	0.303	0.61 (0.49-0.75)	2.64E-06
H5	AC**T**ATATTTCCAGA	0.106	0.130	0.80 (0.61-1.04)	0.10
H6	GTCATGCCTCCAGA	0.047	0.045	1.06 (0.70-1.60)	0.78
H7	ATCA**G**ACCTCCGGG	0.174	0.114	1.65 (1.21-2.11)	7.06E-05
H8	ACCATACTCTTGTG	0.091	0.068	1.36 (0.99-1.86)	0.05

B) 7 SNPs, without recombination

Hap	HAPLOTYPE	Freq cases	Freq controls	OR (95% CI)	P
H1	GTCGTAC	0.214	0.195	1.12 (0.90-1.38)	0.30
H2	AC**T**ATAC	0.210	0.306	0.61 (0.49-0.74)	1.73E-06
H3	GTCATGC	0.194	0.181	1.09 (0.87-1.35)	0.47
H4	AC**T**ATAT	0.106	0.130	0.80 (0.61-1.04)	0.10
H5	ATCA**G**AC	0.176	0.114	1.66 (1.30-2.12)	5.33E-05
H6	ACCATAC	0.091	0.068	1.36 (0.99-1.86)	0.05

OR: odds ratio, CI: confidence interval
