# Supplementary material for: GWAS of Follicular Lymphoma Reveals Allelic Heterogeneity at 6p21.32 and Suggests Shared Genetic Susceptibility with Diffuse Large B-cell Lymphoma
Source: PLoS Genet. 2011 Apr 21;7(4):e1001378. doi: 10.1371/journal.pgen.1001378 (PMC3080853; doi:10.1371/journal.pgen.1001378)
Supplement: Table S10 — Imputation of HLA class II alleles and risk of follicular lymphoma. (0.01 MB PDF) [file pgen.1001378.s016.pdf]

Table S10. Imputation of human leukocyte antigen (HLA) class II alleles and risk of follicular lymphoma (Odds ratio (OR) and trend P-value).

Imputed HLA allele	Frequency in SCALE GWAS samples		Tagging info[c] and Frequency in CEU[3]			Logistic regression		
	Freq cases	Freq controls	HLA freq	r^2	D'	N	OR	P
**HLA-DQA*0101**	0.18	0.13	0.13	1	1	1139	1.40	0.006[a]
**HLA-DQA*0102**	0.28	0.39	0.24	0.88	0.97		Low call rate <90%	
**HLA-DQA*0103**	0.04	0.09	0.06	0.90	1		Low call rate <90%	
**HLA-DQB*0201**	0.21	0.20	0.16	0.96	0.99	1151	1.03	0.81
**HLA-DQB*0301**	0.15	0.15	0.17	1	1		Low call rate <90%	
**HLA-DQB*0302**	0.12	0.13	0.14	1	1	1071	0.94	0.66
**HLA-DQB*0305**	0.06	0.06	0.006	0.50	1	1167	0.93	0.68
**HLA-DQB*0501**	0.17	0.11	0.09	1	1	1170	1.65	7.06E-05[a]
**HLA-DQB*0503**	0.008	0.02	0.04	1	1	1142	0.47	0.08
**HLA-DQB*0602**	0.11	0.14	0.20	0.97	0.99	1159	0.76	0.04
**HLA-DQB*0603**	0.06	0.12	0.05	1	1		Low call rate <90%	
**HLA-DQB*0604**	0.007	0.017	0.02	1	1	1080	0.46	0.10
**HLA-DRB*0101** [b]	0.18	0.11	0.09	1	1	1154	1.66	6.05E-05[a]
**HLA-DRB*0301**	0.12	0.14	0.09	0.87	0.99	1167	0.85	0.22
**HLA-DRB*0401**	0.16	0.13	0.09	0.74	1	1154	1.25	0.08
**HLA-DRB*0402**	0.001	0.003	0.006	0.50	1		Low frequency <1%	
**HLA-DRB*0403**	0.01	0.006	0.02	1	1		Low call rate <90%	
**HLA-DRB*0405**	0.007	0.01	0.006	0.50	0.99	1166	0.69	0.48
**HLA-DRB*0407**	0.018	0.015	0.01	1	1	1090	1.14	0.71
**HLA-DRB*1104**	0.001	0.004	0.006	0.50	1		Low frequency <1%	
**HLA-DRB*1301** [b]	0.04	0.07	0.04	0.88	1	1159	0.59	0.01
**HLA-DRB*1302** [b]	0.03	0.04	0.02	1	1	1132	0.61	0.06
**HLA-DRB*1303** [b]	0.009	0.007	0.01	1	1	1098	1.23	0.69
**HLA-DRB*1401**	NA	0.01	0.03	1	1		Low call rate <90%	
**HLA-DRB*1404**	NA	0.007	0.006	0.50	1		Low call rate <90%	
**HLA-DRB*1501**	0.11	0.14	0.20	0.97	0.99	1159	0.76	0.04

aSignificant association observed at HLA class II alleles tagged by rs10484561, reported in Conde et. al. 2010 Nat Gen [1]
b Previously reported to be associated with FL in Wang et. al. 2010 Blood [4]
cSource: Supplementary Table 3 of deBakker et. al. 2006 Nat Gen [3]
SCALE: Scandinavian lymphoma etiology, GWAS: genome-side association study
