# Supplementary material for: GWAS of Follicular Lymphoma Reveals Allelic Heterogeneity at 6p21.32 and Suggests Shared Genetic Susceptibility with Diffuse Large B-cell Lymphoma
Source: PLoS Genet. 2011 Apr 21;7(4):e1001378. doi: 10.1371/journal.pgen.1001378 (PMC3080853; doi:10.1371/journal.pgen.1001378)
Supplement: Table S11 — Trend p-value of associations of age and sex with main genotypes among controls subjects per study. (0.02 MB PDF) [file pgen.1001378.s017.pdf]

Table S11. Trend p-value of associations of age (≤60 years of age versus >60 years) and sex (male versus female) with main genotypes among controls subjects per study (individual-level data on age and sex were not available for the Yale study or the SF1 study with regard to rs6536942, rs441890 and rs716183)

	Age (<=60 vs>60)						Sex (male vs female)					
	**SCALE1**	**SF**	**BC**	**NCI-SEER**	**NSW**	**Mayo**	**SCALE1**	**SF**	**BC**	**NCI-SEER**	**NSW**	**Mayo**
**rs2647012** chr6:32772436 *HLA-DQB1*	0.96	0.16	0.93	0.13	0.59	0.55	0.74	0.89	0.81	0.80	0.22	0.91
**rs6536942** chr4:167205644 *TLL1*	0.34	-	0.54	0.29	0.15	0.27	0.19	-	0.22	0.22	0.50	0.61
**rs9275574** chr6:33163516 *HLA-DPB1*	0.03	0.84	0.95	0.46	0.76	0.53	0.55	0.38	0.75	0.84	0.07	0.05
**rs441890** chr8:71727221 *LACTB2*	0.37	-	0.47	0.46	0.56	0.35	0.09	-	0.91	0.21	0.71	0.69
**rs716183** chr10:118894485 *VAX1*	0.85	-	0.77	0.03	0.93	0.43	0.61	-	0.20	0.97	0.13	0.89

SCALE: Scandinavian lymphoma etiology, SF: San Francisco, BC: British Columbia, NCI-SEER: National Cancer Institute-Surveillance, Epidemiology and End Results, NSW: New South Wales, Yale: Yale University, Mayo: Mayo Clinic.
