# Supplementary material for: GWAS of Follicular Lymphoma Reveals Allelic Heterogeneity at 6p21.32 and Suggests Shared Genetic Susceptibility with Diffuse Large B-cell Lymphoma
Source: PLoS Genet. 2011 Apr 21;7(4):e1001378. doi: 10.1371/journal.pgen.1001378 (PMC3080853; doi:10.1371/journal.pgen.1001378)
Supplement: Text S1 — Additional description of validation study subjects. (0.04 MB PDF) [file pgen.1001378.s018.pdf]

Text S1: Additional description of validation study subjects

San Francisco (SF1, Stage 2 and SF2, validation in NHL subtypes) study subjects

A population-based case-control study of NHL (2,050 cases, 2,081 controls) was conducted in the San Francisco Bay Area that included incident cases diagnosed from 2001 through 2006. Eligible patients were identified through the Greater Bay Area Cancer Registry rapid case ascertainment system and cancer registry database and met the following criteria at diagnosis: aged 20-85 years, resident in one of the six Bay Area counties and able to complete an interview in English. Controls identified by random digit dialing and random selection from Center for Medicaid & Medicare Services lists were frequency-matched to patients by age in five-year groups, sex and county of residence. Eligibility criteria for controls were the same as for cases with the exception of NHL diagnosis. Blood and/or buccal specimens were collected from eligible cases and controls who participated in the laboratory portion of the study (participation rates, 87% and 89%, respectively). Patient diagnostic pathology materials were re-reviewed by the study's expert pathologist to confirm NHL diagnosis and histology. These samples were used in the previous GWAS studies of FL [1,5].

San Francisco (SF) genotyping (Stage 2)

Genomic DNA from cases and controls was extracted from whole blood using QIAamp DNA Blood Maxi Kit protocol (Qiagen, Valencia, CA) and quantified using PicoGreen dsDNAQuantitation kits (Invitrogen, Carlsbad, CA) according to the manufacturers' specifications. Overall, DNA from 1,577 study participants was genotyped using Illumina HumanCNV370-Duo BeadChip (Illumina, San Diego, CA), which comprises over 370,000 markers, providing 81% of the HapMap variation at r^2>0.8 in a European population (CEU). Genotype clustering was conducted with Illumina Beadstudio software from data files created by an Illumina BeadArray reader. Samples and markers with call rates

below 95% were excluded from further analysis. A total of 339,528 markers were genotyped in 1,568 individuals (236 FL, 221 CLL/SLL, 291 DLBCL, 9 other NHL and 811 controls), and of these, 213 FL, 257 DLBCL and 750 controls were used in Stage 2 and subtype validation of the present study. We further excluded SNPs with call rate <90%, MAF <0.05 and SNPs on sex chromosomes.

NCI-SEER study subjects (Stage 3)

The National Cancer Institute-Surveillance Epidemiology and End Results (NCI-SEER) NHL case-control study included 1,321 newly diagnosed NHL cases identified in four SEER registries (Iowa; Detroit, MI; Los Angeles, CA; and Seattle, WA, USA) at ages 20 to 74 years between July 1998 and June 2000 without evidence of HIV infection [6]. Cases were histologically confirmed by the local diagnosing pathologist and translated to International Classification of Diseases for Oncology (ICD)-O-3/WHO classification [7,8]. Population controls (n=1,057) were identified by random digit dialing (<65 years) and from Medicare eligibility files (≥65 years). Written informed consent was obtained from each participant. Biological samples were obtained for genotyping from 89% of cases and 93% of controls. Of these, genotyping data were obtained from 572 controls and 651 NHL cases (including 176 DLBCL, 171 FL, 72 CLL/SLL and 65 MZL). Approximately 85% of cases and controls were of non-Hispanic European ancestry. Although the numbers of cases and controls were reduced due to depleted DNA from previous efforts, this reduction was consistent by case/control status, and hence the proportion of NHL subtypes was comparable with the original study. This validation cohort was also used in a previous GWAS study of FL [1].

Yale study subjects (Stage 3)

A detailed description of the study population has been reported previously [9,10]. In summary, among female Connecticut residents, a total of 601 histologically confirmed incident NHL cases diagnosed between 1996 and 2000 and 717 population-based controls

aged 21-84 years with no prior history of cancer aside from non-melanoma skin cancer were enrolled and completed in-person interviews. Cases were identified through the Yale Cancer Center's Rapid Case Ascertainment Shared Resource, histologically confirmed by two independent study pathologists and classified into NHL subtypes according to the WHO classification system [7]. Control women were identified via random-digit dialing (for those aged less than 65 years) or random selection from Centers for Medicare and Medicaid Services records (for those aged 65 or older) and were frequency matched to the cases on age. About 76.7% of the cases and 74.6% of the controls provided blood samples and about 11.0% of the cases and 10.4% of the controls provided buccal cell samples for genotyping, including 98 FL, 125 DLBCL, 38 CLL/SLL, 28 MZL and 460 controls. DNA was extracted from blood and buccal cell using phenol-chloroform extraction. The genotyping data are missing for some cases and controls for three reasons: the amount of DNA was too low for some samples (buccal cell samples were excluded from genotyping because of very low DNA amounts and some blood samples had inadequate DNA for genotyped as well), samples failed to amplify, or samples amplified but their genotype could not be determined due to ambiguous results. This validation cohort was used in a previous GWAS study of FL [1].

NSW study subjects (Stage 3)

Details of the process and criteria for subject selection have been fully described [11]. Patients notified to the New South Wales (NSW) Central Cancer Registry with newly diagnosed NHL between January 1, 2000 and August 31, 2001 who were 20–74 years of age and residing in NSW or the Australian Capital Territory (ACT) were potentially eligible to be cases; those with a history of transplantation or HIV infection were excluded. Pathology reports for consenting cases were reviewed by one anatomical pathologist to assign a cell phenotype and WHO (ICD-O-3) code [7,8]. Controls were randomly selected from the NSW and ACT electoral rolls to match approximately the expected distributions of cases with

respect to age, sex and residence (NSW or ACT). Eighty five percent of cases invited to participate did so, as did 61% of controls. Of all eligible and participating subjects (687 cases and 694 controls), 597 cases and 525 controls provided blood, and genomic DNA was extracted successfully from buffy coat specimens for 584 cases and 518 controls. After restricting the study sample to participants of European ethnicity (95% of study subjects) with sufficient DNA available, 373 cases (including 161 FL, 124 DLBCL, 15 CLL/SLL and 40 MZL) and 398 controls were genotyped for this project. The study was approved by all relevant ethical committees and written informed consent was obtained from all participants. This validation cohort was also used in the previous GWAS study [1].

BC study subjects (Stage 3)

This study has been described previously [12]. All HIV-negative NHL cases diagnosed in British Columbia, Canada, from March 2000 to February 2004, residing in the Greater Vancouver Regional District and greater Victoria and aged 20 to 79 years, were invited to participate. Cases were reviewed and coded using the WHO classification by an expert lymphoma pathologist [7,8]. Population controls were identified from the Client Registry of the British Columbia Ministry of Health and were frequency matched to cases by sex, age, and area of residence in a 1:1 ratio. Blood was obtained from approximately 90% of study participants; 10% provided saliva or a mouthwash sample. 25% of DNA samples that were genotyped were whole-genome amplified (WGA) (Qiagen RepliG); inclusion of samples pre- and post-WGA demonstrated 97% concordance between genotypes generated with WGA vs. genomic DNA. For this analysis, results are reported for the 174 FL cases and 610 controls who self-reported that their grandparents were all of European ancestry. All studies were approved by the local ethical committees and written informed consent was obtained from all participants. This validation cohort was also used in the previous GWAS study [1].

NCI-SEER, NSW, Yale and BC genotyping (Stage 3)

Assays were obtained from the Assays-on-Demand service (Applied Biosystems). TaqMan reactions were carried out in 5 or 15 µL volumes containing 10-20ng DNA according to the manufacturer's protocols. Fluorescence data was obtained in the ABI PRISM 7900HT, after 10 min at 95 ºC, followed by 40 cycles of 92 ºC for 15 s and 60 ºC for 1 min. SDS2.2 software (Applied Biosystems) was used to call genotypes.

Mayo Clinic study subjects and genotyping (Stage 3)

This study was reviewed and approved by the Human Subjects Institutional Review Board at the Mayo Clinic, and all participants provided written, informed consent. Full details of this on-going, clinic-based case-control study conducted at the Mayo Clinic in Rochester, Minnesota have been previously reported [12]; this report is based on cases and controls enrolled from September 1, 2002 to February 28, 2008. Briefly, we offered enrollment to consecutive patients with newly diagnosed (within nine months of first diagnosis), histologically-confirmed Hodgkin lymphoma or NHL (including the subtype of chronic lymphocytic leukemia/small lymphocytic lymphoma) who were aged 20 years or older, HIV-negative, and residing in Minnesota, Iowa or Wisconsin at the time of diagnosis. All cases were reviewed and confirmed by a hematopathologist, and classified according to the WHO criteria[2]. Of the 1798 eligible cases, 1236 (69%) participated. Clinic-based controls were selected from patients visiting the Mayo Clinic Department of Medicine for a pre-scheduled general medical exam. Eligibility requirements for controls included being 20 years or older and a resident of Minnesota, Iowa or Wisconsin; patients were excluded if they had prior diagnoses of lymphoma, leukemia, or HIV infection. Controls were randomly selected and frequency matched to the cases by 5-year age group, gender, and region of residence. Of the 1899 eligible controls, 1315 (69%) participated. All participants were

asked to complete a self-administered risk-factor questionnaire and provide a blood sample. DNA was extracted using a standard procedure (Gentra Inc., Minneapolis, MN).

Genotyping was conducted as part of a larger study using a custom Illumina GoldenGate [13] 1536 SNP OPA that included the 6 SNPs for this study. Of the 1536 SNPs genotyped, 1459 passed all quality controls (including no clustering issues; call rates >95%). Of the 3565 unique samples genotyped (this number includes subjects not in the case-control study), 3377 samples with a call rate >90% were retained. The concordance rate across 201,509 duplicate genotypes was 99.6%. For this analysis, only European subjects from the case-control study were included, leaving a total of 1089 NHL cases (including 246 FL, 193 DLBCL, 327 CLL/SLL and 61 MZL) and 1233 controls.

Controlling for population stratification in the validation studies

The possibility of population stratification affecting the results of the validation studies (Stages 2 and 3, and validation in NHL subtypes) has been thoroughly explored in earlier studies on the same cohorts [1,13]. In all studies, only subjects of self-reported European ancestry were included in the analyses. In the SF1 and SF2 studies, multi-dimensional scaling (MDS) was used to assess population substructure and non-European subjects were removed after inspection of the MDS plot [1]. The inflation factor (λ) was estimated to be about 1.04, suggesting minimal stratification that could have biased the results [1]. In the Mayo study, 1000 independent (r^2<0.25) and randomly selected SNPs that were genotyped for an earlier genetic study [13] were assessed using the program STRUCTURE, and no evidence of population stratification was found [13]. For the remaining studies, population substructure has been assessed using MDS (BC; 42 ancestry informative markers, λ=1.007) or PCA (NCI-SEER, NSW, Yale; 1597 genotyped SNPs) analyses, and no evidence of population stratification was found [1].

References

1. Conde L, Halperin E, Brown KM, Smedby KE, Rothman N, et al. (2010) Genome-wide association study of follicular lymphoma identifies a risk locus at 6p21.32. Nat Genet 42: 661-664.
2. Stephens M, Smith NJ, Donnelly P (2001) A new statistical method for haplotype reconstruction from population data. Am J Hum Genet 68: 978-989.
3. de Bakker PI, McVean G, Sabeti PC, Miretti MM, Green T, et al. (2006) A high-resolution HLA and SNP haplotype map for disease association studies in the extended human MHC. Nat Genet 38: 1166-1172.
4. Wang SS, Abdou AM, Morton LM, Thomas R, Cerhan JR, et al. (2010) Human leukocyte antigen class I and II alleles in non-Hodgkin lymphoma etiology. Blood 115: 4820-4823.
5. Skibola CF, Bracci PM, Halperin E, Conde L, Craig DW, et al. (2009) Genetic variants at 6p21.33 are associated with susceptibility to follicular lymphoma. Nat Genet 41: 873-875.
6. Wang SS, Cerhan JR, Hartge P, Davis S, Cozen W, et al. (2006) Common genetic variants in proinflammatory and other immunoregulatory genes and risk for non-Hodgkin lymphoma. Cancer Res 66: 9771-9780.
7. Jaffe ES, Harris NL, Stein H, Vardiman J (2008) WHO classification of hematopoetic and lymphoproliferative malignancies: IARC press.
8. Turner JJ, Hughes AM, Kricker A, Milliken S, Grulich A, et al. (2004) Use of the WHO lymphoma classification in a population-based epidemiological study. Ann Oncol 15: 631-637.
9. Zhang Y, Holford TR, Leaderer B, Boyle P, Zahm SH, et al. (2004) Hair-coloring product use and risk of non-Hodgkin's lymphoma: a population-based case-control study in Connecticut. Am J Epidemiol 159: 148-154.
10. Zhang Y, Lan Q, Rothman N, Zhu Y, Zahm SH, et al. (2005) A putative exonic splicing polymorphism in the BCL6 gene and the risk of non-Hodgkin lymphoma. J Natl Cancer Inst 97: 1616-1618.
11. Hughes AM, Armstrong BK, Vajdic CM, Turner J, Grulich A, et al. (2004) Pigmentary characteristics, sun sensitivity and non-Hodgkin lymphoma. Int J Cancer 110: 429-434.
12. Spinelli JJ, Ng CH, Weber JP, Connors JM, Gascoyne RD, et al. (2007) Organochlorines and risk of non-Hodgkin lymphoma. Int J Cancer 121: 2767-2775.
13. Cerhan JR, Ansell SM, Fredericksen ZS, Kay NE, Liebow M, et al. (2007) Genetic variation in 1253 immune and inflammation genes and risk of non-Hodgkin lymphoma. Blood 110: 4455-4463.
